# Supplementary material for: Ribonucleotide reductase subunit switching in hepatoblastoma drug response and relapse
Source: Commun Biol. 2023 Mar 8;6:249. doi: 10.1038/s42003-023-04630-7 (PMC9992519; doi:10.1038/s42003-023-04630-7)

Supplementary Table 1. IC_{50} values of the RNR-manipulated HepG2 and HB214 cells and *P* values of their comparisons.

			Gemcitabine	Cisplatin	Vincristine	Triapine	MK1775	Sorafenib	Doxorubicin	SN38
HepG2	IC50 (mM)	TdT	2.719	6.960	0.426	2.289	0.699	3.870	0.167	0.040
		RRM2^{OE}	11.740	18.600	4.771	4.108	2.465	6.013	0.434	0.088
	P value	TdT vs. RRM2^{OE}	0.0031	<0.0001	0.0002	0.0044	0.0266	0.0003	0.0002	0.2635
	IC50 (mM)	TdT	2.719	6.960	0.426	2.289	0.699	3.870	0.162	0.040
		RRM2B^{OE}	9.865	19.560	5.916	3.270	1.042	5.744	0.348	0.093
	P value	TdT vs. RRM2B^{OE}	0.0044	<0.0001	<0.0001	0.1508	0.49	0.0002	0.022	0.1161
	IC50 (mM)	Ctrl	1.354	11.270	1.305	3.906	1.801	4.620	0.243	0.281
		RRM2B^{KO1}	1.811	11.220	1.595	3.985	2.411	5.082	0.275	0.433
	P value	Ctrl vs. RRM2B^{KO1}	0.5028	0.9365	0.6754	0.8666	0.3995	0.1985	0.4519	0.294
	IC50 (mM)	Ctrl	1.354	11.270	1.305	3.906	1.801	4.620	0.247	0.281
		RRM2B^{KO2}	0.457	11.210	0.522	3.501	3.510	6.416	0.251	0.268
	P value	Ctrl vs. RRM2B^{KO2}	0.0164	0.934	0.0737	0.4085	0.0538	<.0001	0.9283	0.9174
	IC50 (mM)	RRM2B^{KO1}	1.811	11.220	1.595	3.985	2.411	5.082	0.275	0.433
		RRM2B$^{KO1/Res}$	undetermined	19.730	10.270	10.300	3.882	5.903	0.518	0.204
	P value	RRM2B$^{KO1\ vs.}$ RRM2B$^{KO1/Res}$	undetermined	<.0001	<.0001	<.0001	0.1539	0.0159	<.0001	0.0455
	IC50 (mM)	RRM2B^{KO2}	0.457	11.210	0.522	0.501	3.510	6.416	0.251	0.268
		RRM2B$^{KO2/Res}$	2.333	17.500	2.008	7.082	2.939	5.383	0.472	0.151
	P value	RRM2B$^{KO2\ vs.}$ RRM2B$^{KO2/Res}$	0.0005	<.0001	0.0097	<.0001	0.5846	0.0006	0.0007	0.2176
HB214	IC50 (mM)	Ctrl	5.386	17.090	0.006	2.841	0.312	4.654	0.173	0.008
		RRM2B^{KO}	5.993	15.180	0.041	3.600	1.573	4.509	0.334	0.035
	P value	Ctrl vs. RRM2B^{KO}	0.7328	0.0639	0.0081	0.2855	0.0002	0.7204	0.0104	0.0022

Supplementary Table 2. ZIP synergy scores of in vitro HepG2 and HB214 combinatorial drug treatments.

Cell Line	Drug 1	Drug 2	ZIP Score
**HepG2**	Triapine	Cisplatin	-3.905
	Triapine	Doxorubicin	-5.167
	Triapine	Vincrisitine	-2.478
	MK1775	Triapine	-5.174
**HB214**	Triapine	Cisplatin	-8.898
	Triapine	Doxorubicin	-5.979
	Triapine	Vincristine	1.602
	Triapine	SN38	**2.816**
	MK1775	Triapine	0.01
	MK1775	SN38	**7.307**

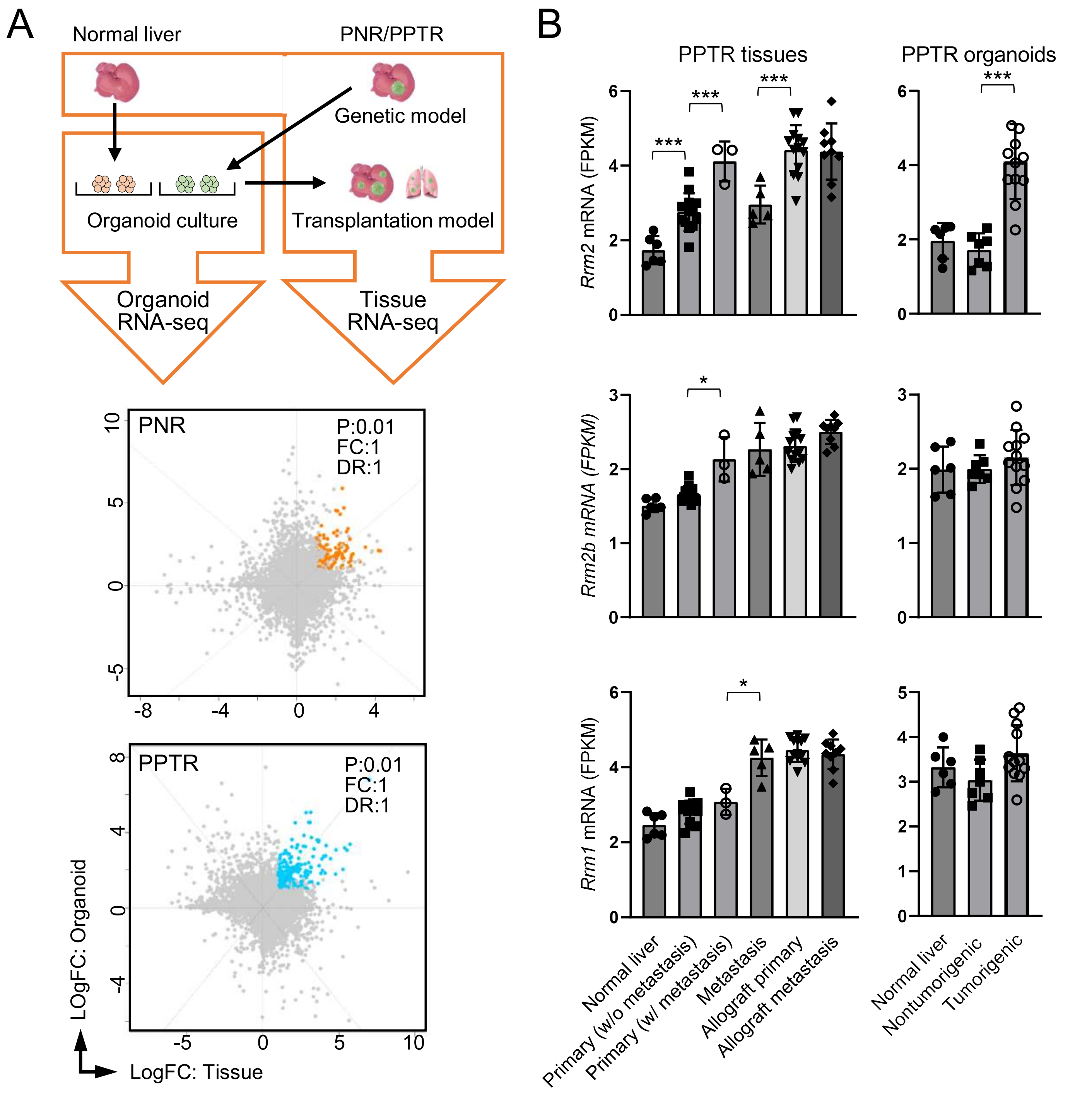

Supplementary Figure 1. RRM2 is associated with the disease progression in PPTR mouse model of HCC.

(A) Flow chart for the comparative transcriptomic analysis of the tumors and organoids from the PNR and PPTR mouse models.

(B) Quantitative comparison of the expression of three RNR subunits in PPTR tumor tissues (N = 6, 13, 3, 5, 13, 9, respectively, for the six groups presented) and organoids (N = 6, 7, 12, respective, for the three groups presented) in the indicated groups.

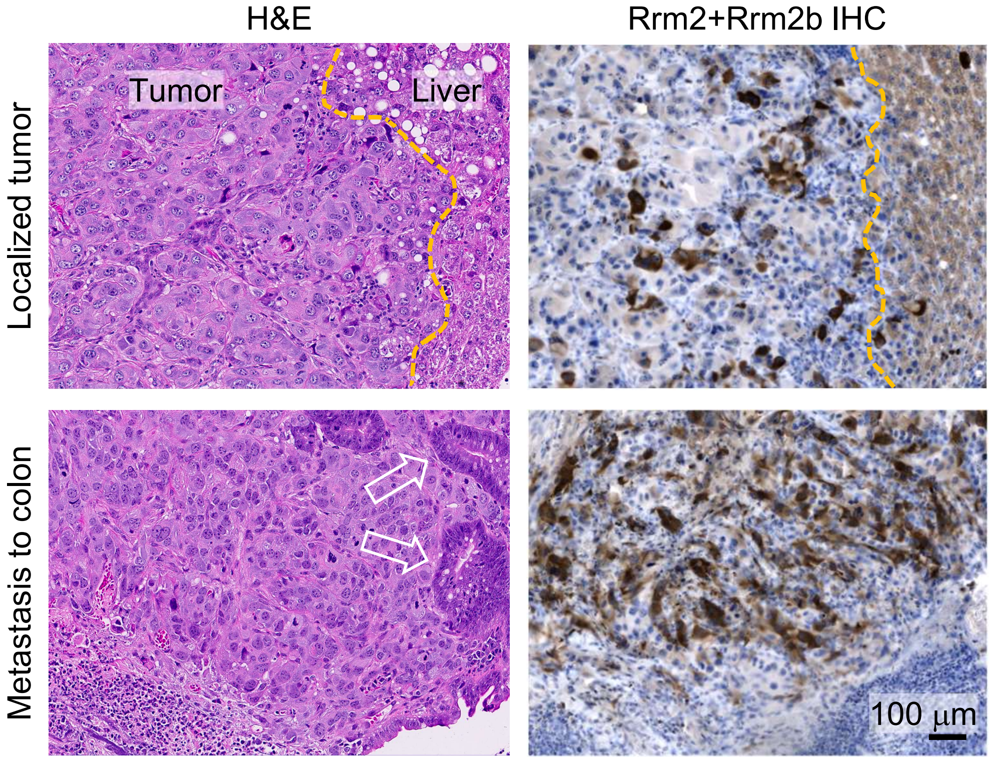

Supplementary Figure 2. Combined RRM2 and RRM2B levels are elevated in metastatic PNR tumors compared to localized tumors.

H&E (left) and RRM2+RRM2B IHC staining (right) on serial sections of a localized and a metastatic PNR tumor. Dotted lines: tumor border; arrows: colonic polyps embedded in the tumor. All images share the same 100 μm scale bar.

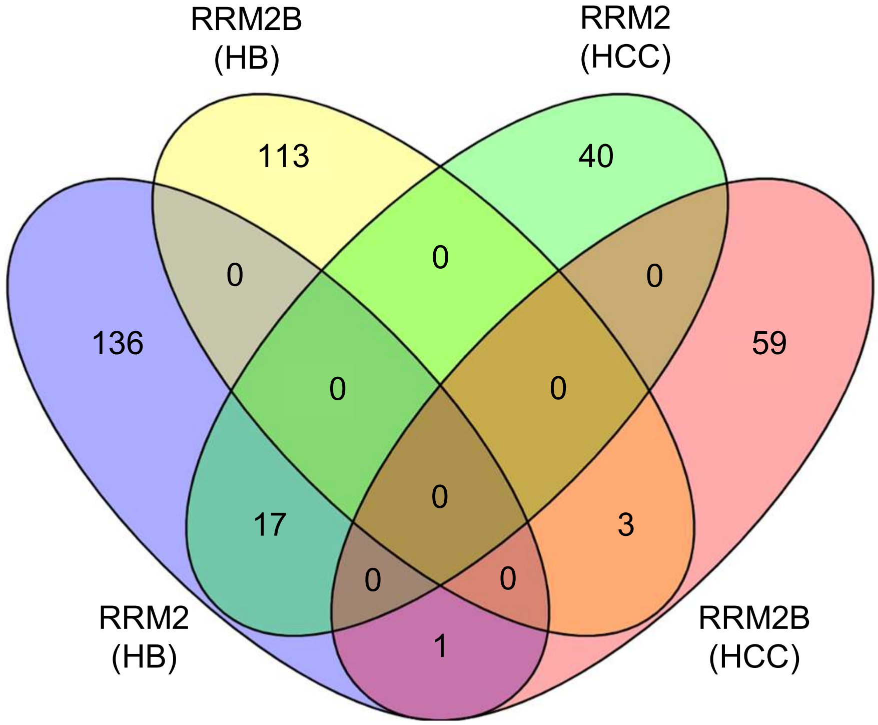

Supplementary Figure 3. Limited overlap of the *RRM2* and *RRM2B* hub genes between HB and HCC patient tumors.

Venn plot showing the overlap of the four indicated *RRM2* and *RRM2B* hub gene lists identified in HB and HCC patient tumors.

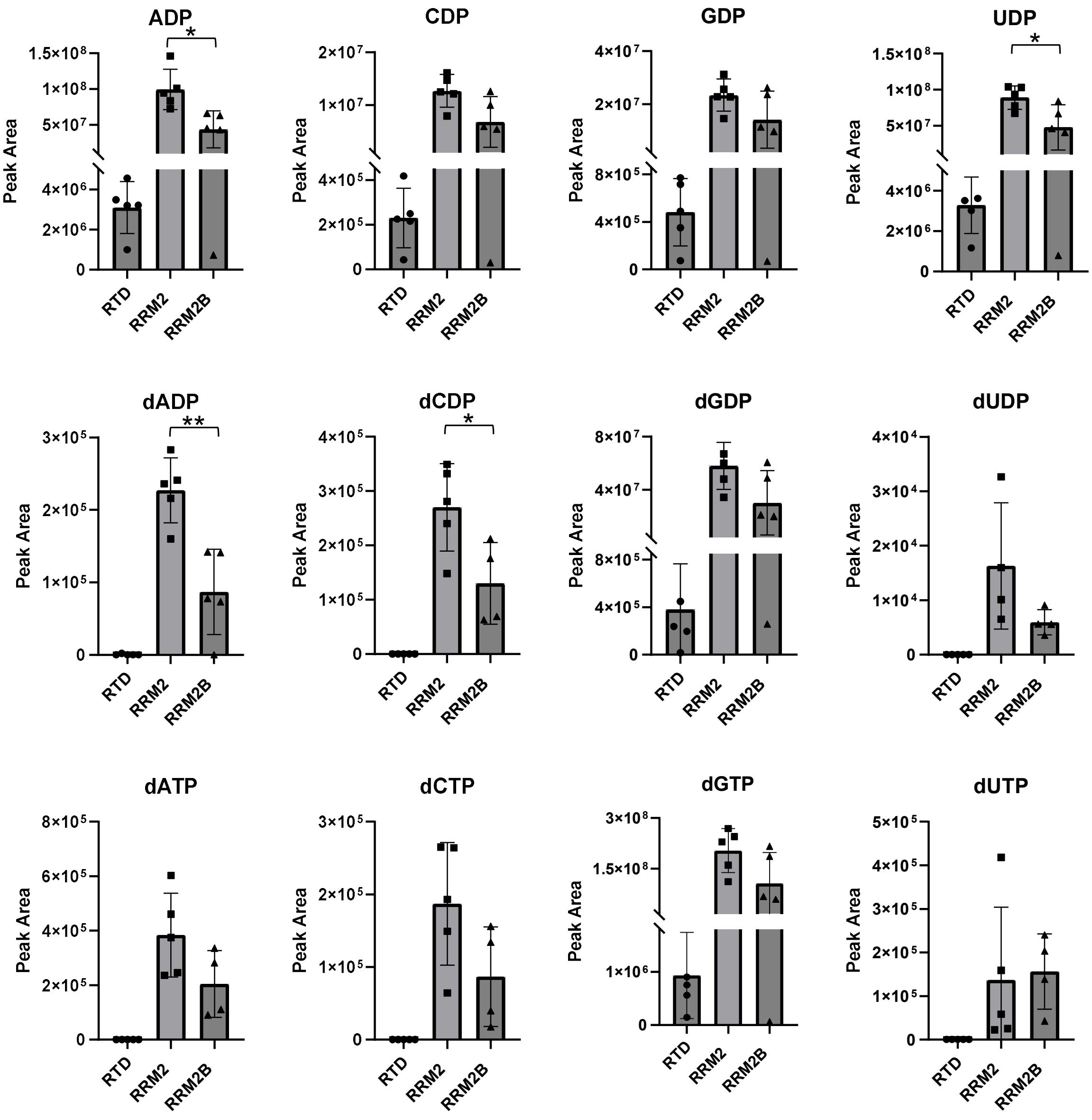

Supplementary Figure 4. RRM2 has higher RNR enzymatic activity than <u>RRM2B</u> in HepG2 cells.

Quantitative analysis of nucleotide levels in *tdT*, *RRM2*^{OE}, and <u>*RRM2B*</u>^{OE} HepG2 cells using targeted liquid chromatography/mass spectrometry (biological replicates: n=5 per group).

A

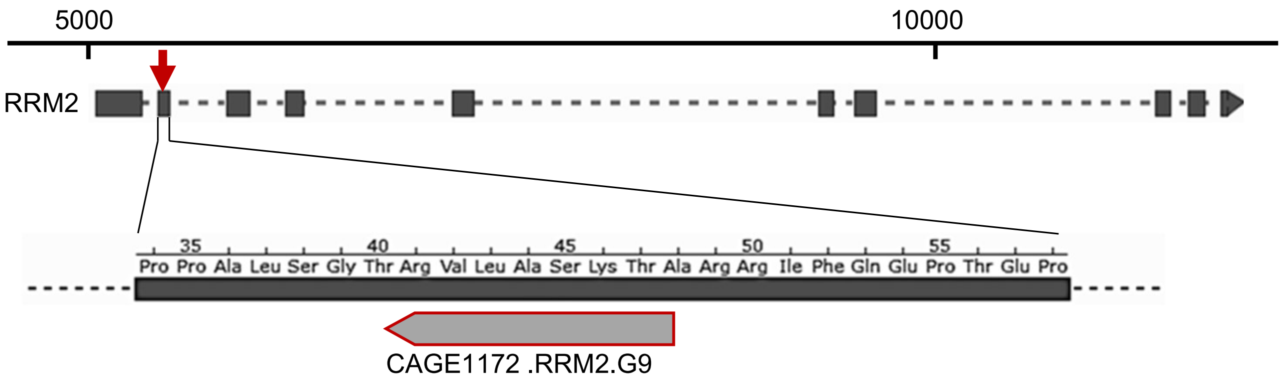

B

Indel Ranking	bp change	Clone%
**#1**	**0**	**13.9%**
#2	-1	9.2%
**#3**	**-18**	**7.2%**
**#4**	**-6**	**5.2%**
**#5**	**-12**	**4.5%**
**#6**	**-21**	**4.5%**
**#7**	**-24**	**4.2%**
#8	-13	3.7%
**#9**	**-3**	**3.7%**
#10	1	3.0%
	Total indel	86.10%
	**Total in-frame indel**	**43.2%**

C

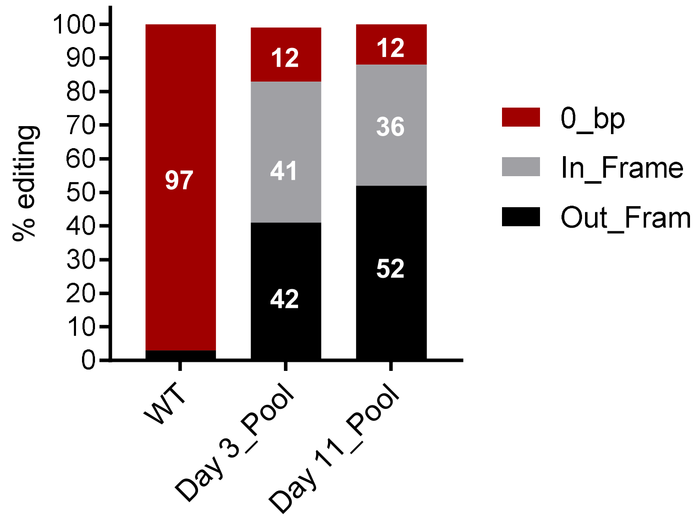

Supplementary Figure 5. RRM2 is an essential gene in HepG2 cells.

(A) The position and sequence of *RRM2* guide RNA.

(B) Indels after attempted knockout of *RRM2* in *RRM2B*^{OE} HepG2 cells.

(C) Quantitative analysis of *RRM2* indels composition on Day 3 and Day 11 of in *RRM2B*^{OE} HepG2 cells.

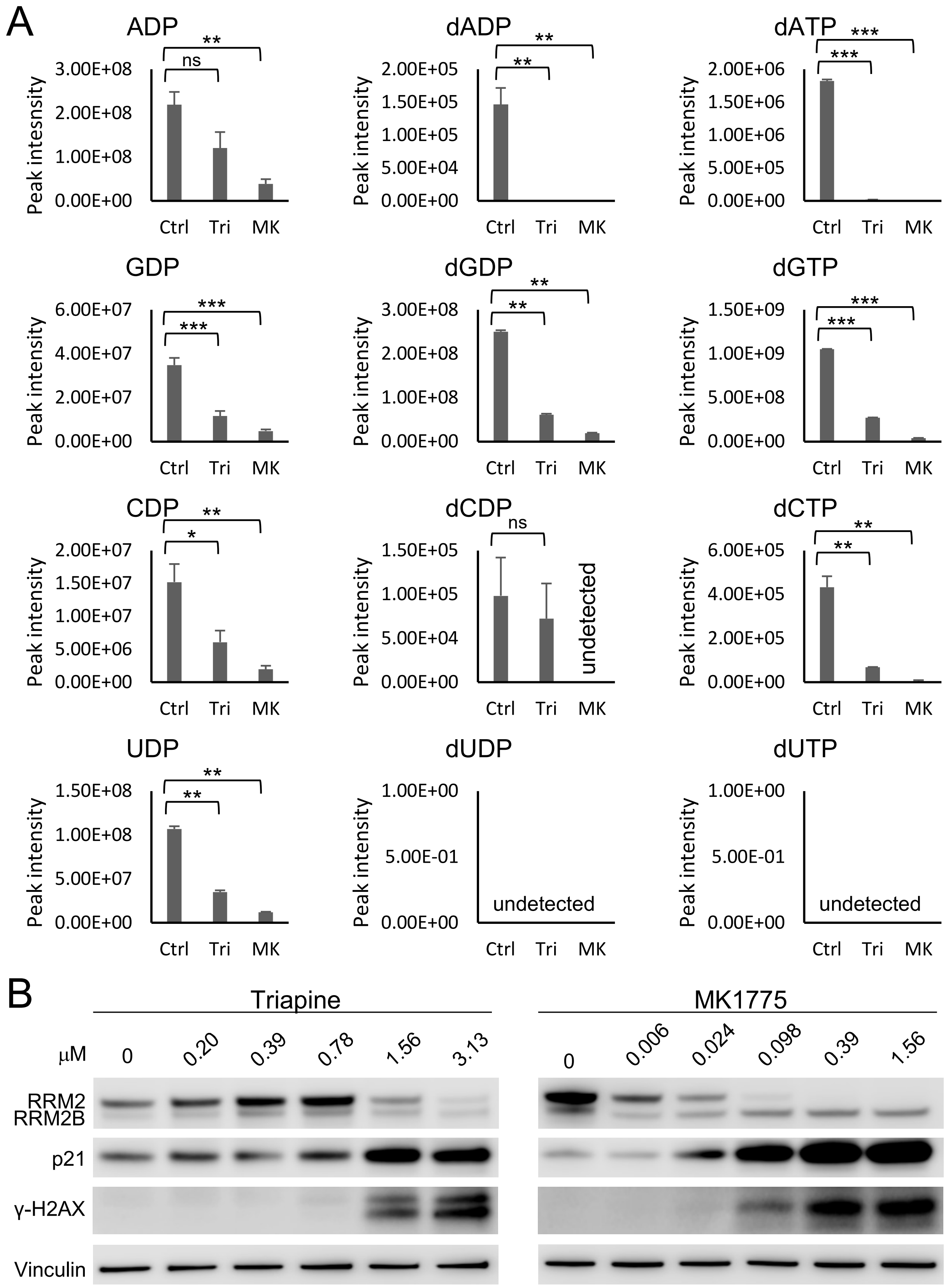

Supplementary Figure 6. RRM2 inhibition in HepG2 cells leads to nucleotide reduction, cell cycle arrest and DNA damage response.

(A) Quantitative analysis of nucleotide levels in HepG2 cells treated with control (ctrl, DMSO), triapine (Tri, 3.125 μM), and MK1775 (MK, 0.39 μM) via targeted liquid chromatography/mass spectrometry (biological replicates: n=5 per group).

(B) Immunoblotting of the indicated proteins in HepG2 cells treated with triapine and MK1775 at the indicated concentration.

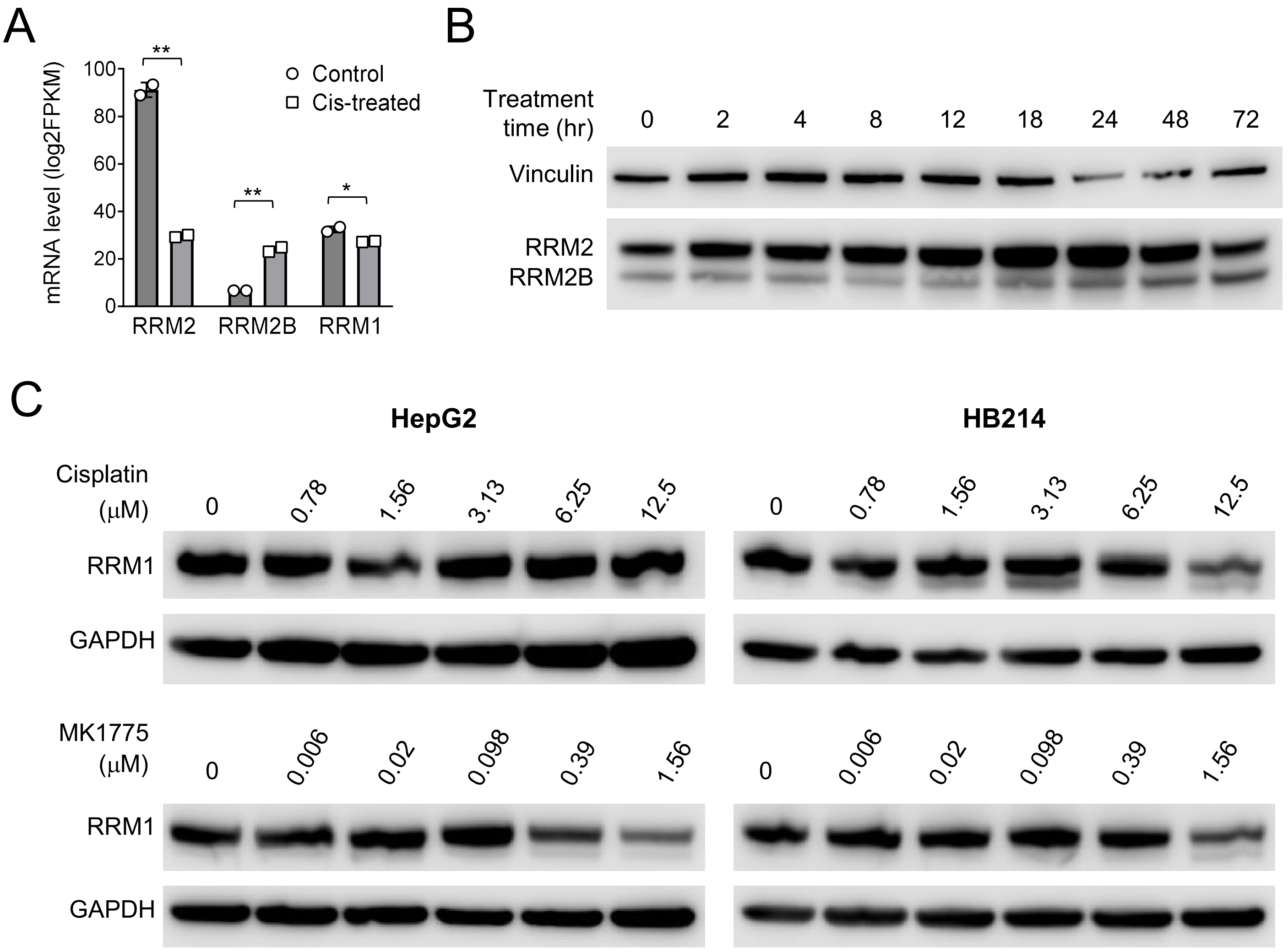

Supplementary Figure 7. Drug treatment induces different gene expression changes to the three RNR subunits in HepG2 cells.

(A) RNR subunits mRNA levels detected by RNAseq in HepG2 cells treated with cisplatin (biological replicates: n=2 per group).

(B) A time-course study of RRM2 and RRM2B protein levels in cis-treated HepG2 cells by immunoblotting.

(C) RRM1 immunoblotting in HepG2 and HB214 cells treated with cisplatin and MK1775.

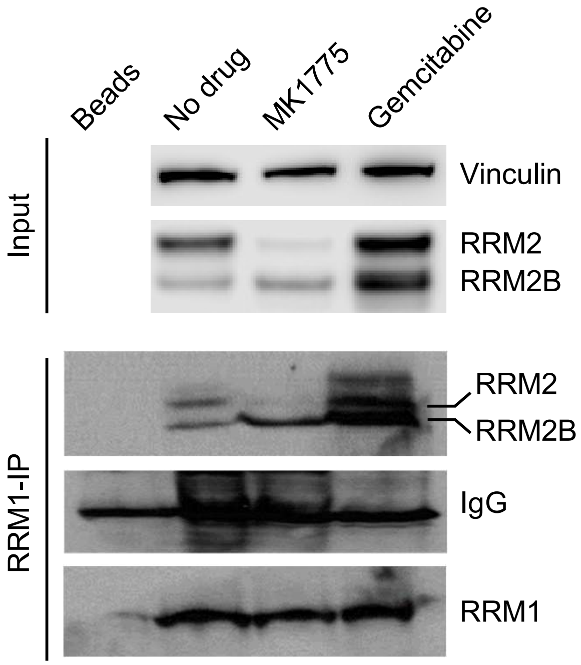

Supplementary Figure 8. RRM2B-RRM1 complex is the dominant RNR complex in drug-treated HepG2 cells.

RRM1 co-IP assay using MK1775- and gemcitabine-treated HepG2 cells.

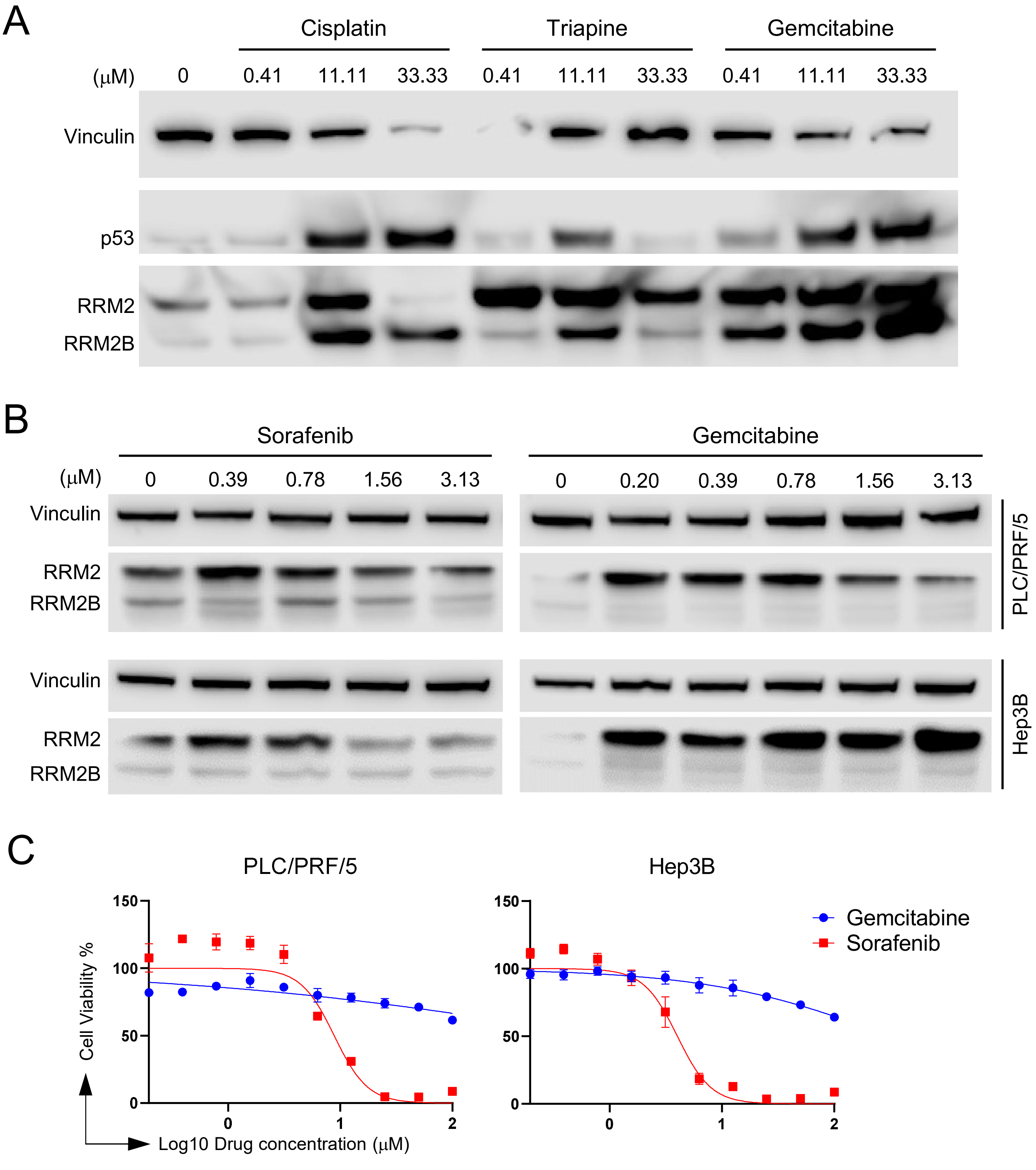

Supplementary Figure 9. Drug-induced RRM2B upregulation in HB cells coincides with p53 induction.

(A) Immunoblot of p53, RRM2 and RRM2B in HepG2 cells treated with the indicated drugs.

(B) RRM2 and RRM2B immunoblotting in two *TP53*-mutant HCC cell lines, PLC/PRF/5 and Hep3B, treated with sorafenib and gemcitabine.

(C) Dose response curves of PLC/PRF/5 and Hep3B cells treated with sorafenib and gemcitabine. All drug curves represent three technical replicates. All assays were biologically repeated for three times.

A

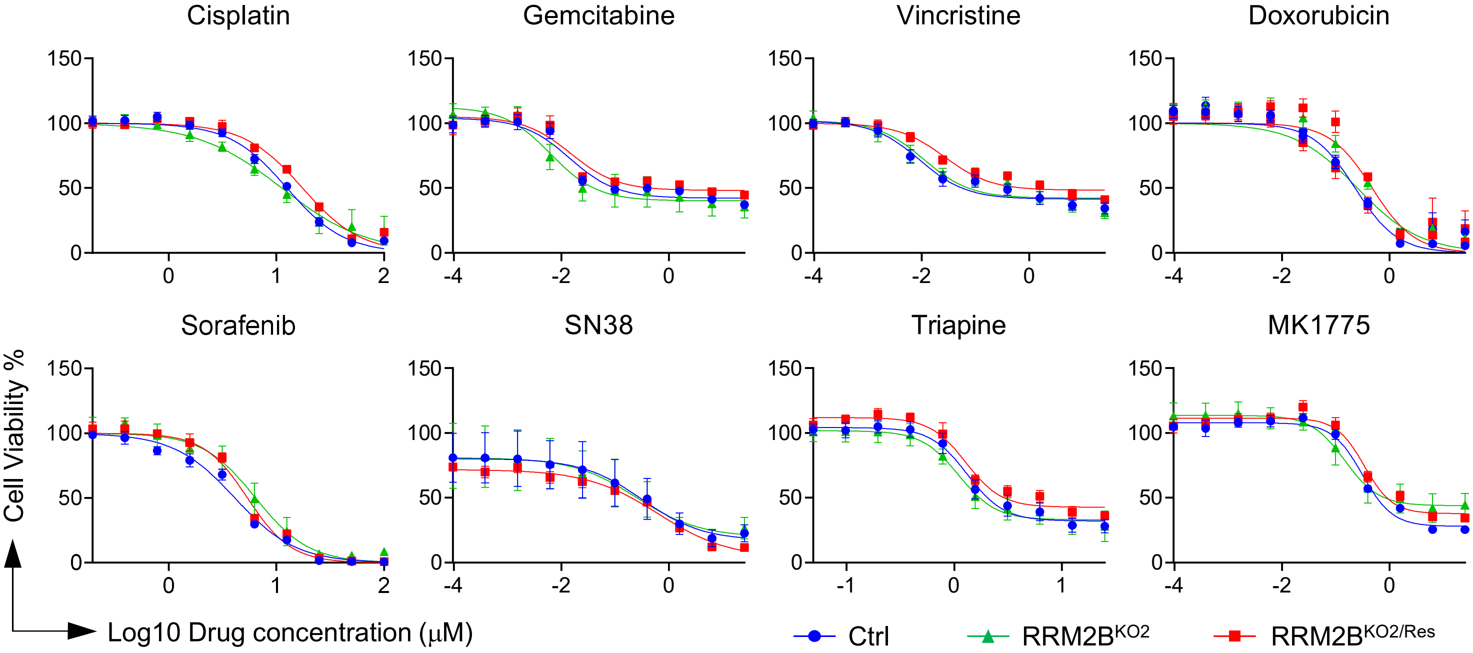

B

	Cisplatin	Gemcitabine	Vincristine	Doxorubicin	Sorafenib	SN38	Triapine	MK1775
IC_{50}: Ctrl	11.27	1.354	1.305	0.2427	4.62	0.2805	3.906	1.801
IC_{50}: RRM2B^{KO2}	11.21	0.4569	0.5218	0.2508	6.416	0.2676	3.501	3.51
IC_{50}: RRM2B$^{KO2/Res}$	17.5	2.333	2.008	0.4716	5.383	0.1512	7.082	2.939
P value: RRM2B^{KO2} vs. ctrl	0.934	0.0164	0.0737	0.9283	<.0001	0.9174	0.4085	0.0538
P value: RRM2B$^{KO2/Res}$ vs. RRM2B^{KO2}	<.0001	0.0005	0.0097	0.0007	0.0006	0.2176	<.0001	0.5846

Supplementary Figure 10. Drug response curves of control, *RRM2B*^{KO2} and *RRM2B*$^{KO2/Res}$ HepG2 cells.

(A) The dose-response curves of control (wildtype), *RRM2B*^{KO2} and *RRM2B* $^{KO2/Res}$ HepG2 cells to the indicated drugs. All drug curves represent three technical replicates. All assays were biologically repeated for three times.

(B) List of the drug IC_{50} values and their comparisons between control (wildtype), *RRM2B*^{KO2} and *RRM2B* $^{KO2/Res}$ HepG2 cells. Extra Sum of Square F test. A *P* value < 0.05 is considered statistically significant.

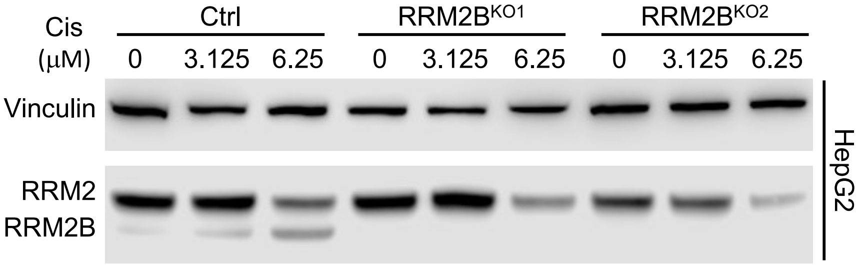

Supplementary Figure 11. Confirmation of the lack of *RRM2B* induction in *RRM2B*^{KO} HepG2 cells.

RRM2 and RRM2B immunoblotting in untreated and cisplatin-treated *RRM2B*^{KO} HepG2 cells.

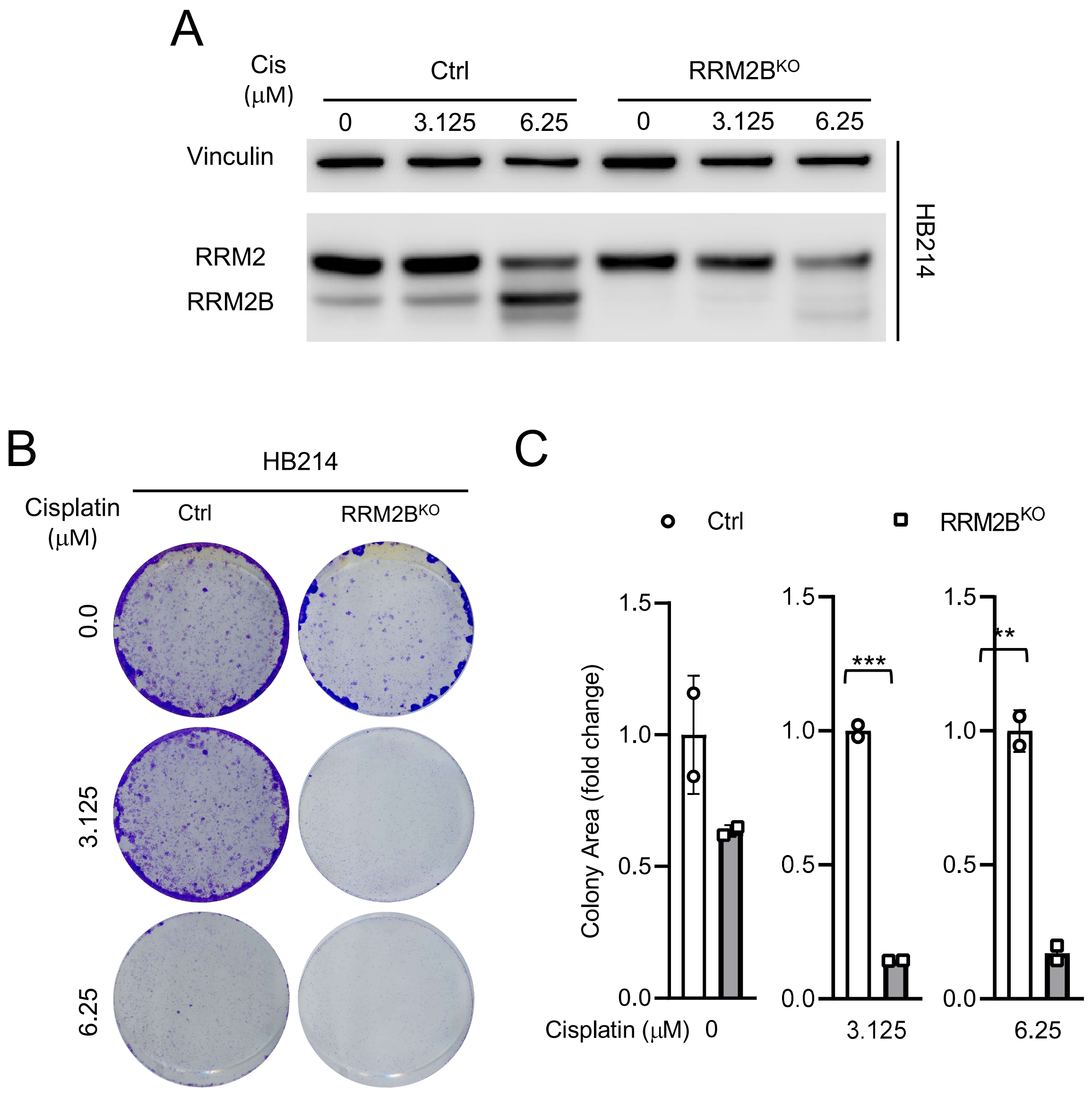

Supplementary Figure 12. RRM2B supports post-drug treatment recovery of HB214 cells in vitro.

(A) RRM2 and RRM2B immunoblotting in untreated and cis-treated *RRM2B*^{KO} HB214 cells.

(B) 12-day colony formation assay of the control and *RRM2B*^{KO} HB214 cells post the indicated treatment.

(C) Quantitative analysis of area occupied by cells in **(B)** (biological replicates, n = 2 per group).

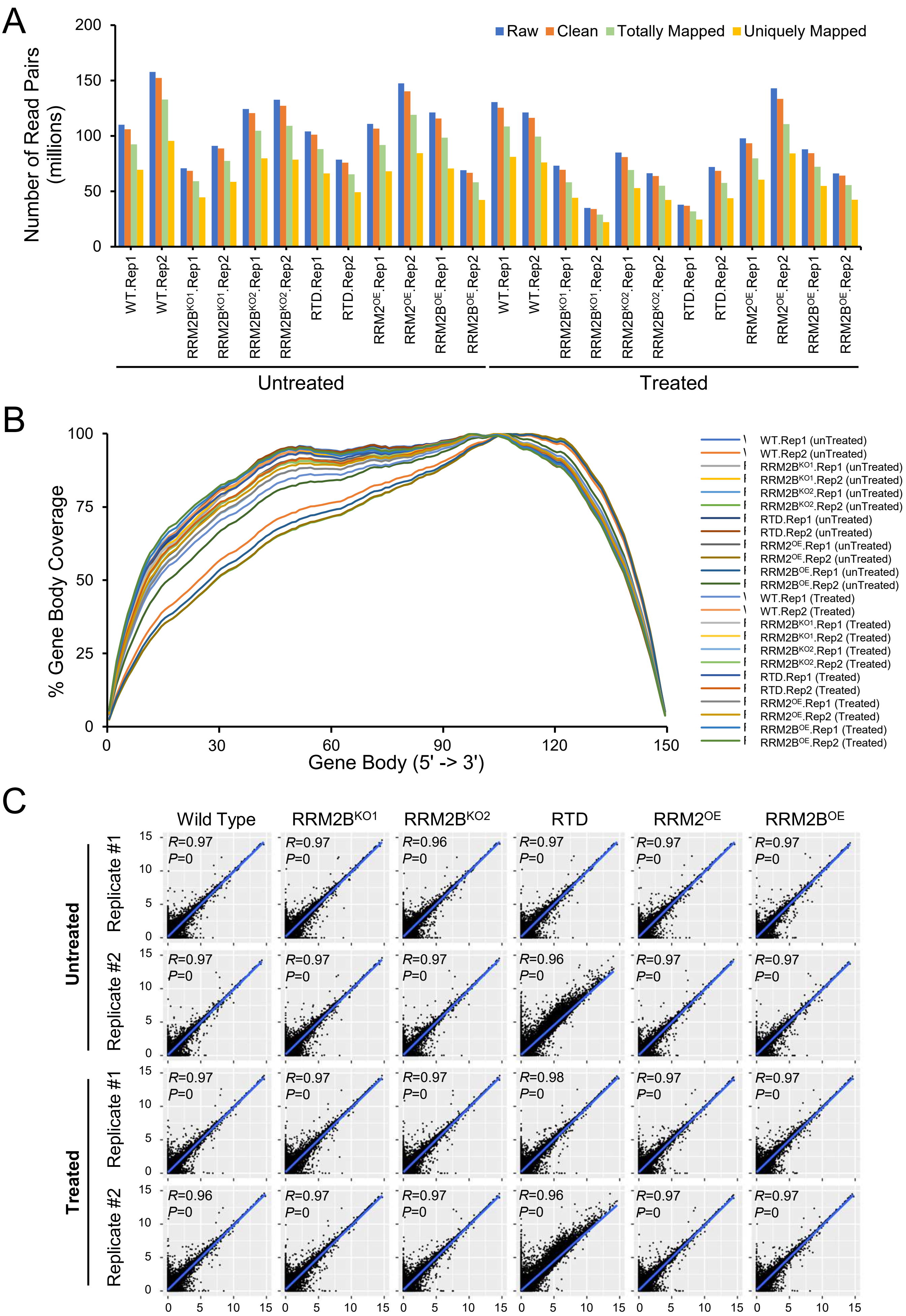
A
Number of Read Pairs (millions)
Raw
Clean
Totally Mapped
Uniquely Mapped
WT.Rep1
WT.Rep2
RRM2B^KO1.Rep1
RRM2B^KO1.Rep2
RRM2B^KO2.Rep1
RRM2B^KO2.Rep2
RTD.Rep1
RTD.Rep2
RRM2^OE.Rep1
RRM2^OE.Rep2
RRM2B^OE.Rep1
RRM2B^OE.Rep2
Untreated
Treated
B
% Gene Body Coverage
Gene Body (5' -> 3')
WT.Rep1 (unTreated)
WT.Rep2 (unTreated)
RRM2B^KO1.Rep1 (unTreated)
RRM2B^KO1.Rep2 (unTreated)
RRM2B^KO2.Rep1 (unTreated)
RRM2B^KO2.Rep2 (unTreated)
RTD.Rep1 (unTreated)
RTD.Rep2 (unTreated)
RRM2^OE.Rep1 (unTreated)
RRM2^OE.Rep2 (unTreated)
RRM2B^OE.Rep1 (unTreated)
RRM2B^OE.Rep2 (unTreated)
WT.Rep1 (Treated)
WT.Rep2 (Treated)
RRM2B^KO1.Rep1 (Treated)
RRM2B^KO1.Rep2 (Treated)
RRM2B^KO2.Rep1 (Treated)
RRM2B^KO2.Rep2 (Treated)
RTD.Rep1 (Treated)
RTD.Rep2 (Treated)
RRM2^OE.Rep1 (Treated)
RRM2^OE.Rep2 (Treated)
RRM2B^OE.Rep1 (Treated)
RRM2B^OE.Rep2 (Treated)
C
Wild Type
RRM2B^KO1
RRM2B^KO2
RTD
RRM2^OE
RRM2B^OE
Untreated
Treated
Replicate #1
Replicate #2
R=0.97 P=0
R=0.96 P=0
R=0.98 P=0

Supplementary Figure 13. Quality assessment of HepG2 cell transcriptomic analyses.

(A) Bar plot showing the alignment statistics of the RNA-Seq data.
(B) The gene body coverage statistics at the resolution of 150 bins per transcript.
(C) The accuracy evaluation of gene expression quantification by RSEM. The Spearman correlation coefficient and P-value were calculated from the genes co-identified by RSEM and Salmon using the stats R package (v3.6.1).

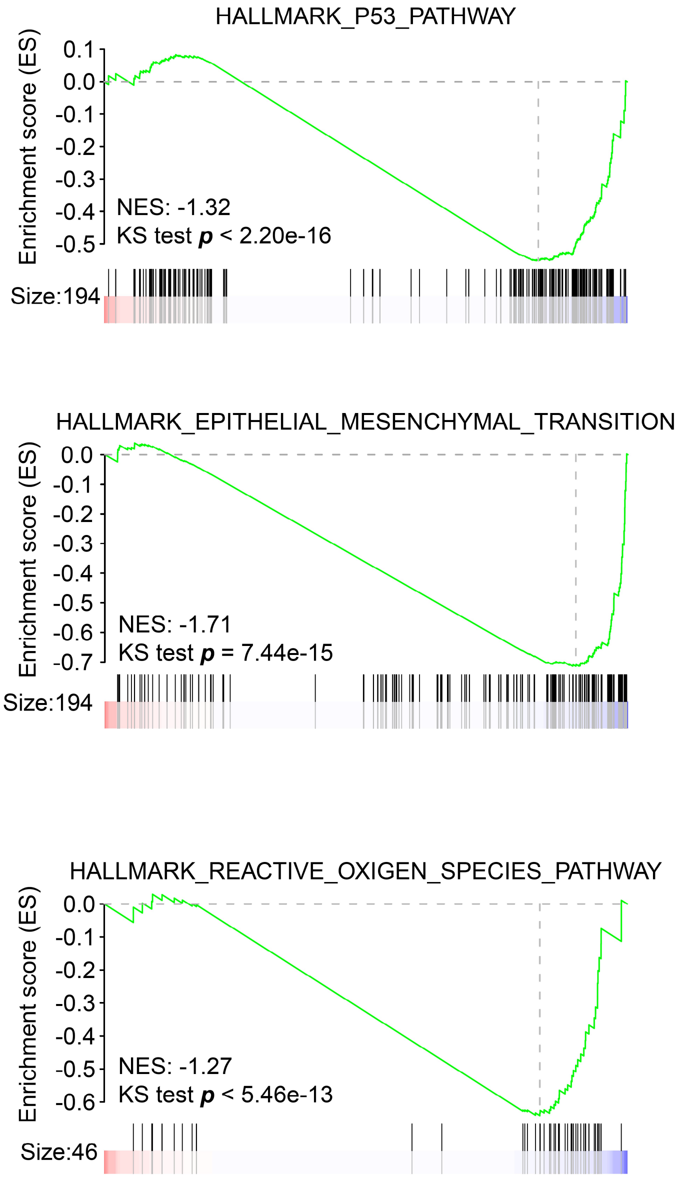

Supplementary Figure 14. GSEA plots of six main RRM2B-involved pathways.

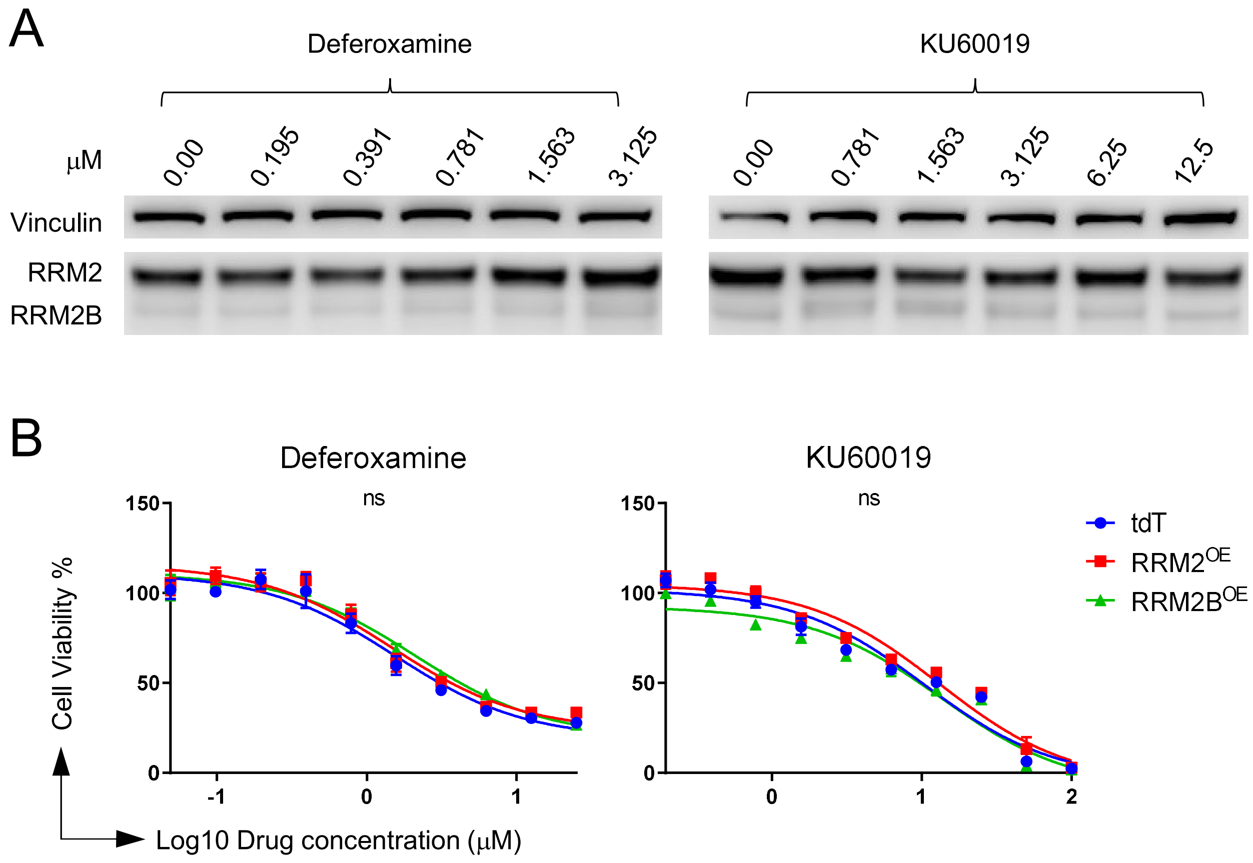

Supplementary Figure 15. Two putative RRM2B inhibitors deferoxamine and KU60019 have no RRM2B-specific inhibition in HepG2 cells.

(A) Immunoblot for RRM2 and RRM2B in HepG2 cells treated with deferoxamine and KU60019.

(B) Dose response curves of *tdT*, *RRM2*^{OE}, and *RRM2B*^{OE} HepG2 cells to deferoxamine and KU60019. Extra Sum of Square F test; ns: not significant (*P* value > 0.05). All drug curves represent three technical replicates. All assays were biologically repeated for three times.

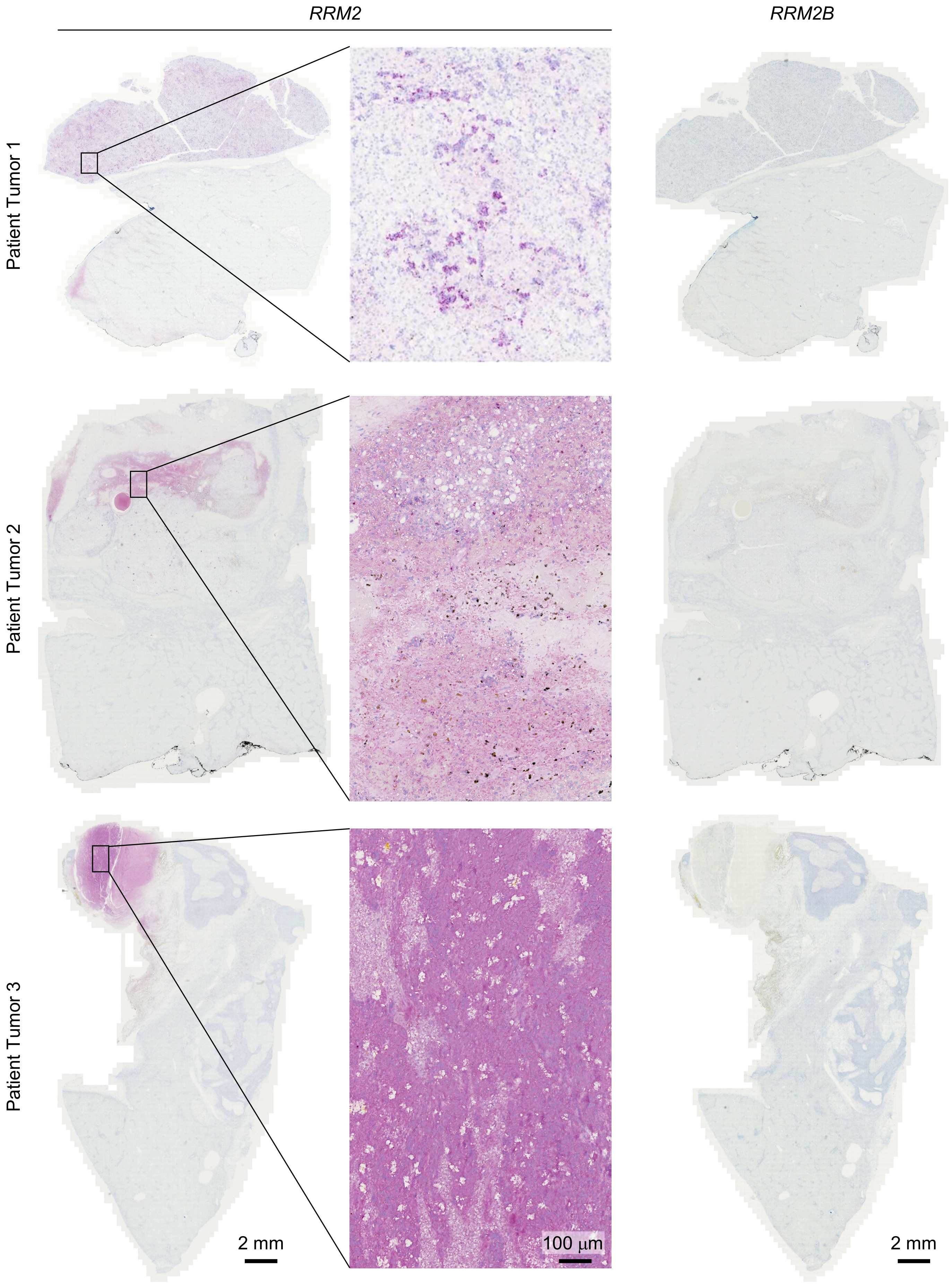

Supplementary Figure 16. *RRM2* and *RRM2B* RNAscope staining on three primary HB patient tumors. Images on the same column share the same scale bar.

Supplementary Figure 17. Uncropped and unedited blot/gel images in the indicated figures.

Figure 3A

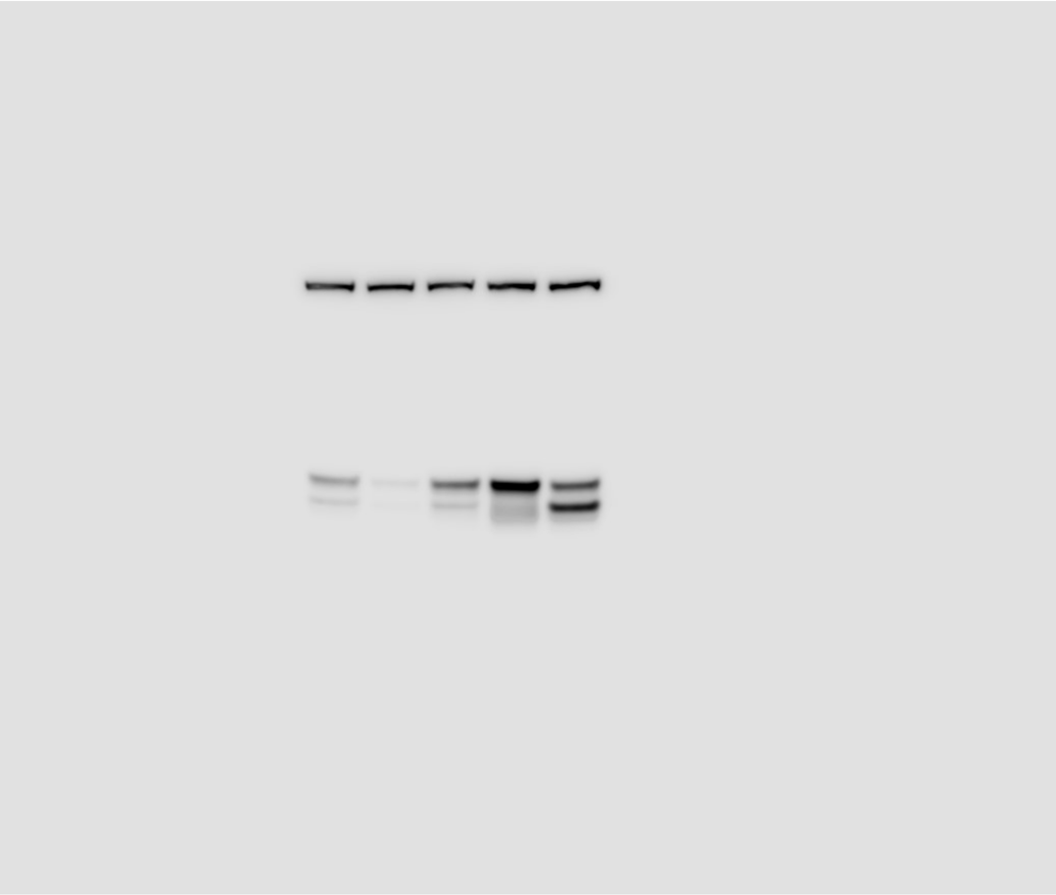

Figure 3C

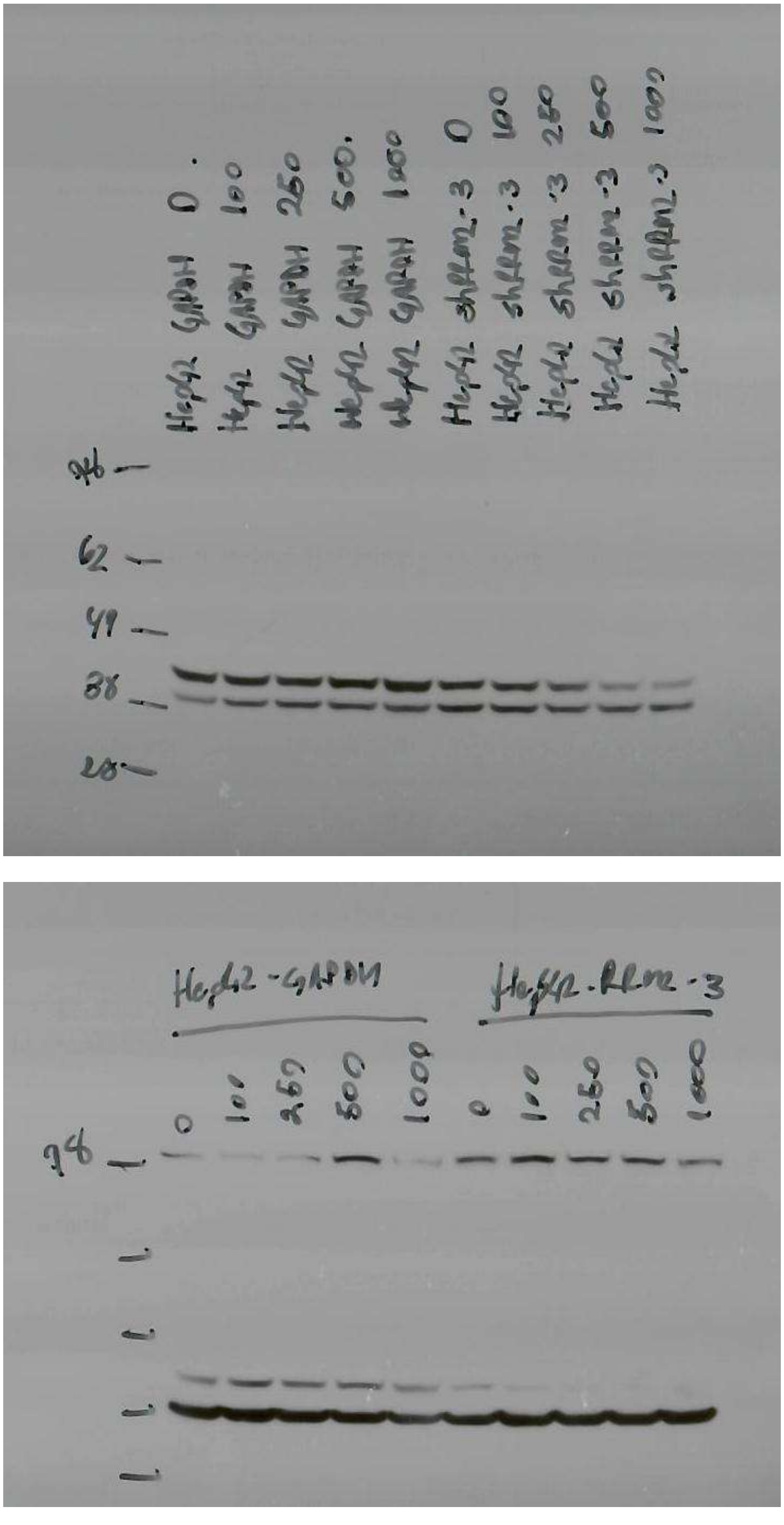

Figure 3E

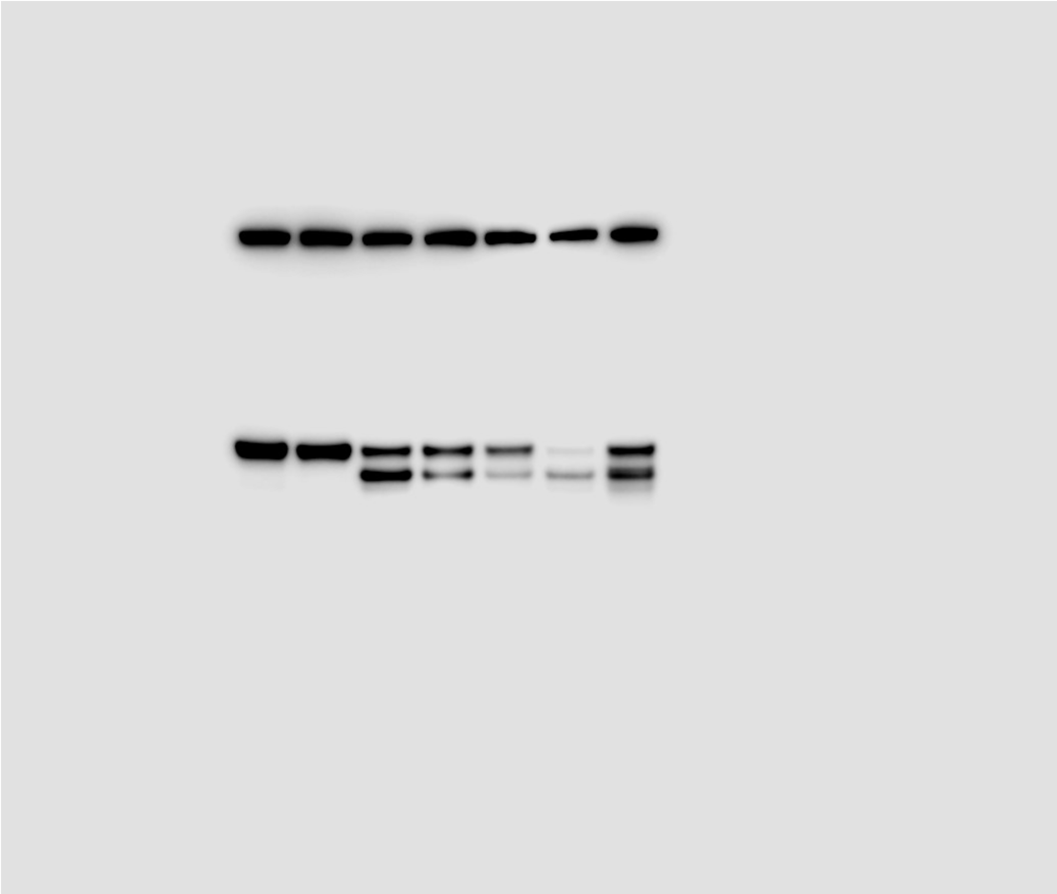

Figure 4B

Cisplatin Treatment

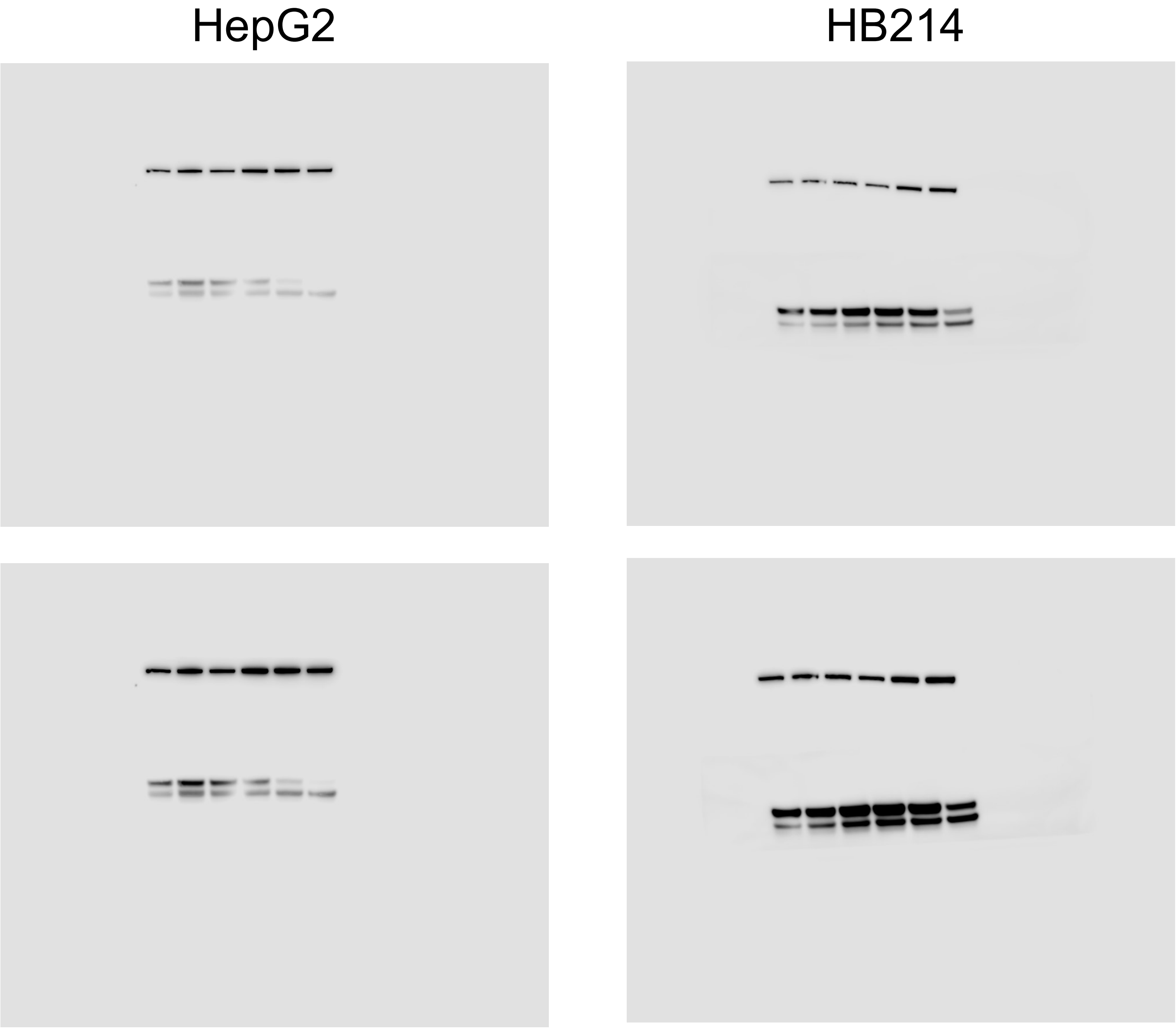

Figure 4B

Gemcitabine Treatment

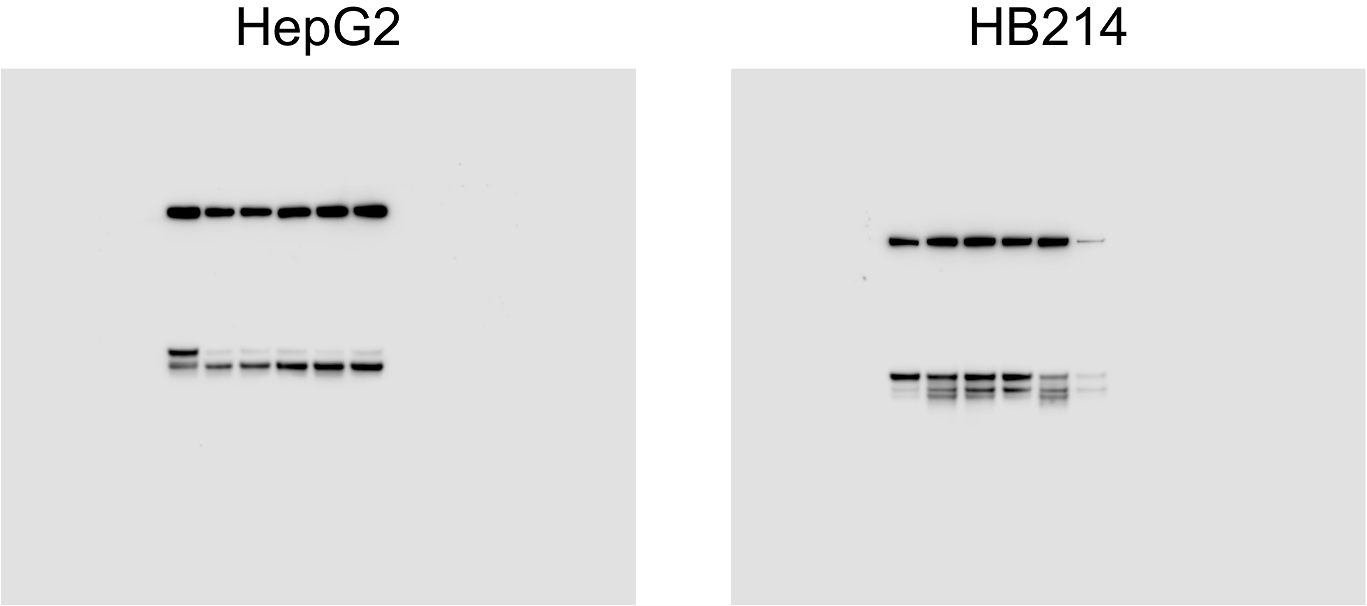

Figure 4B

Vincristine Treatment

HepG2 HB214

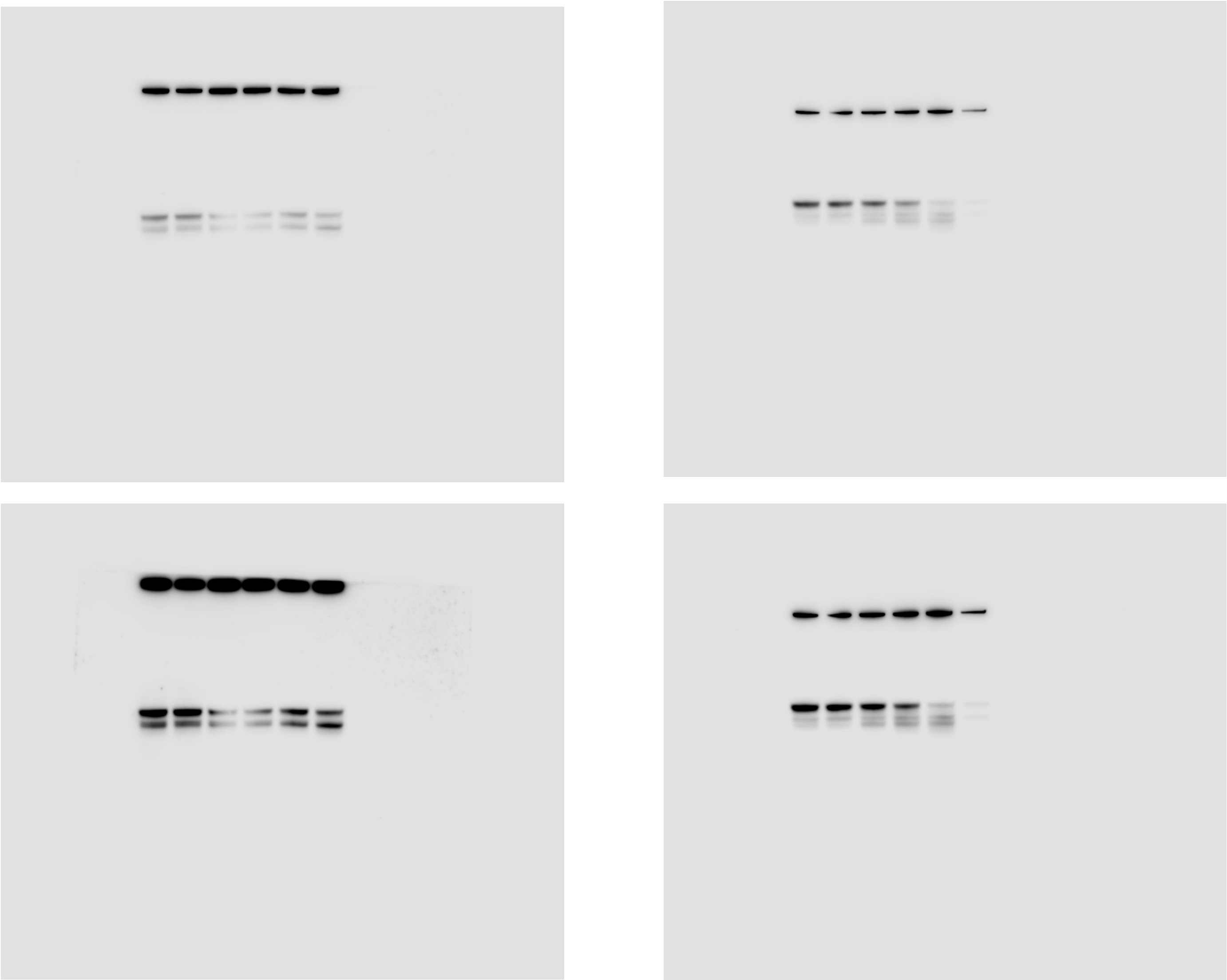

Figure 4B

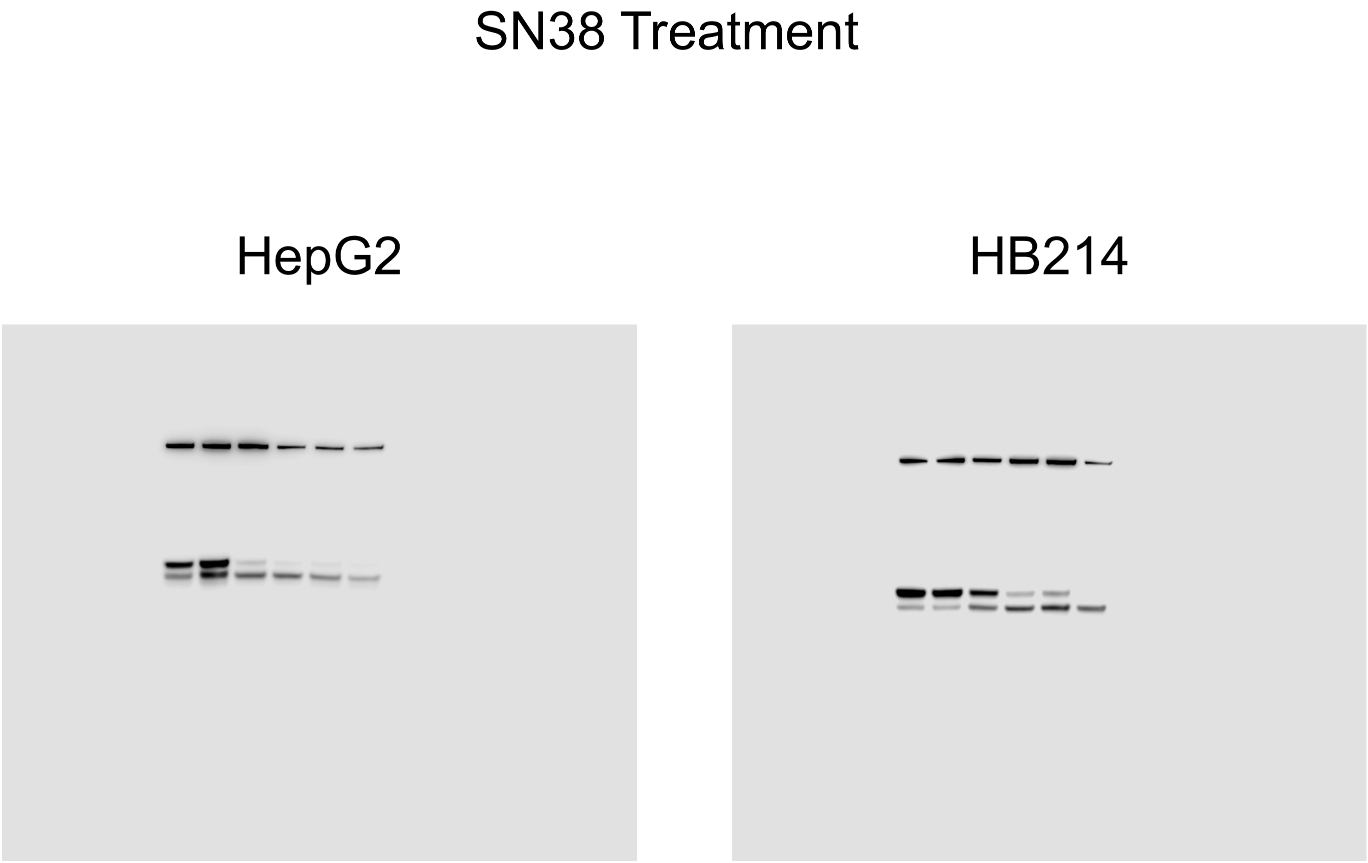

Figure 4B

MK1775 Treatment

HepG2

HB214

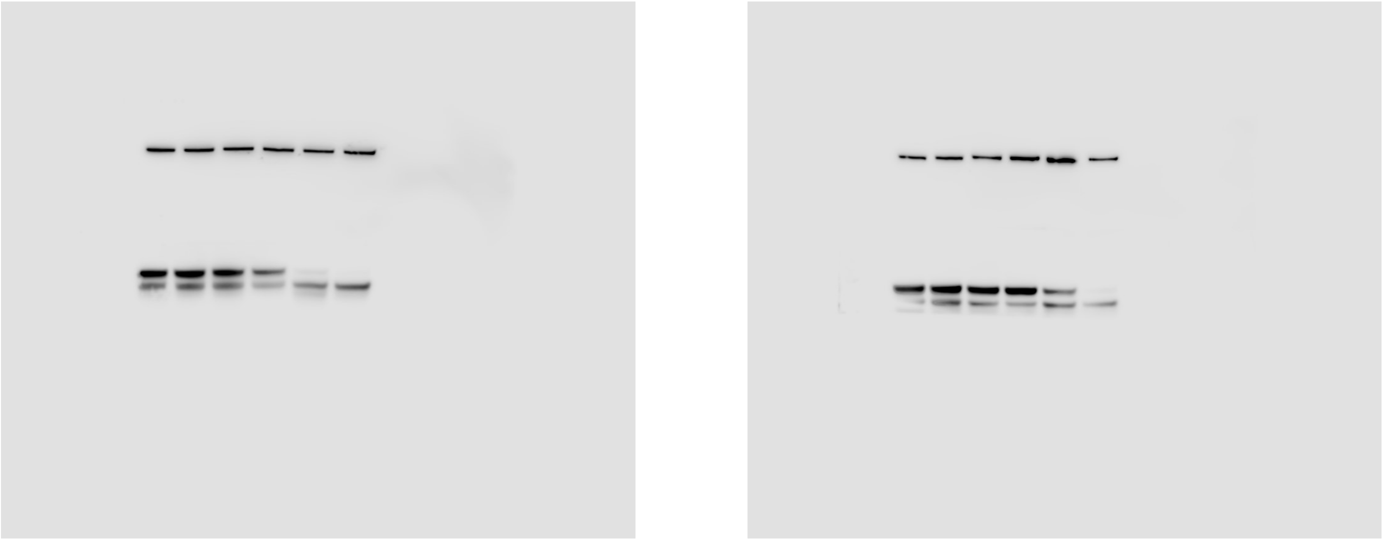

Figure 4B

Triapine Treatment

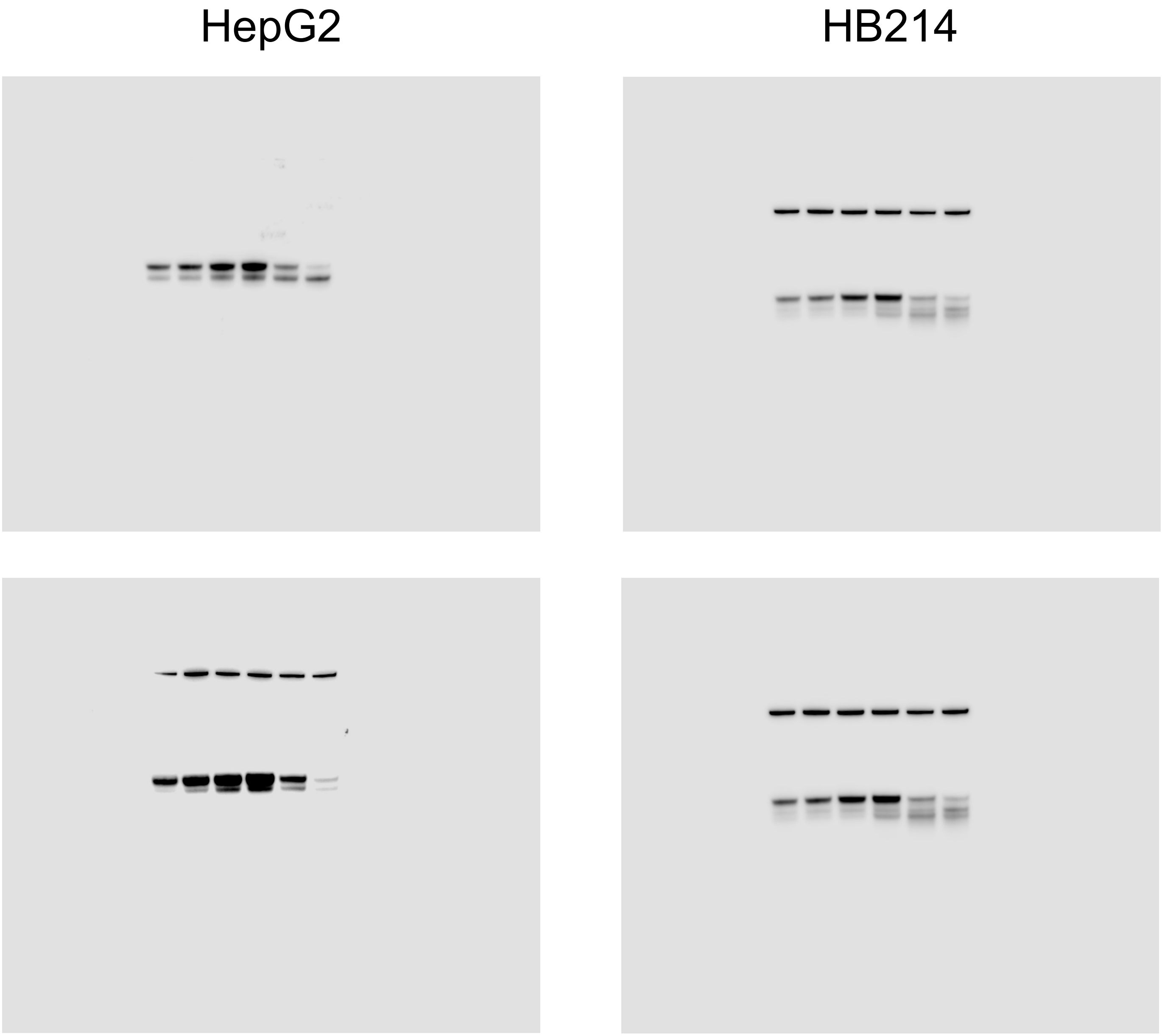

Figure 6E

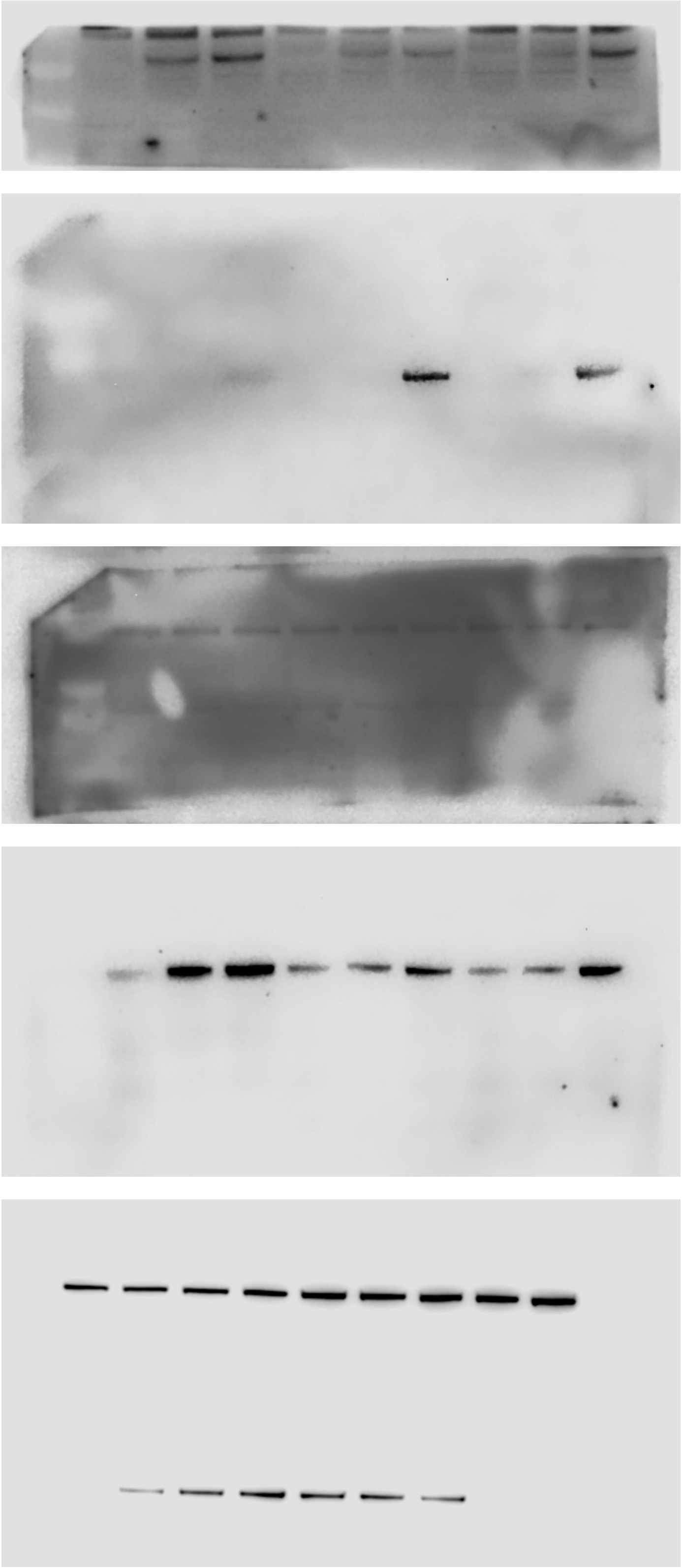

Figure 7A

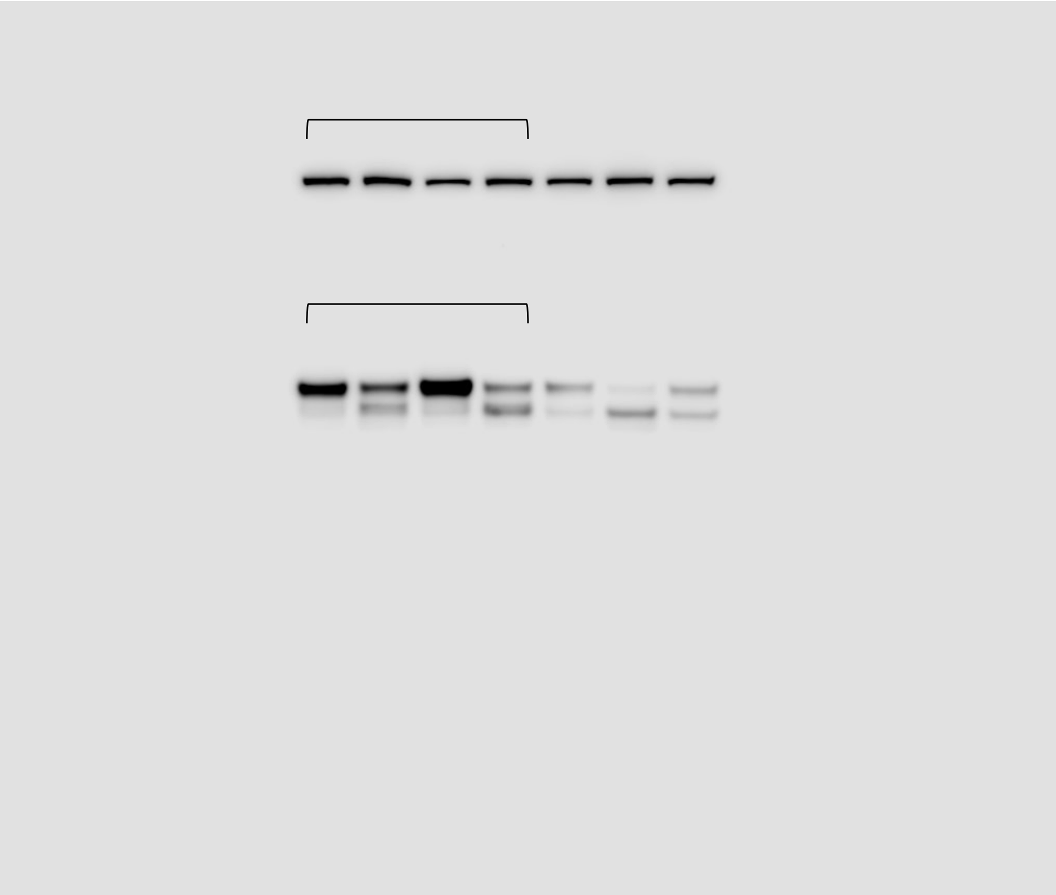

Figure 7F

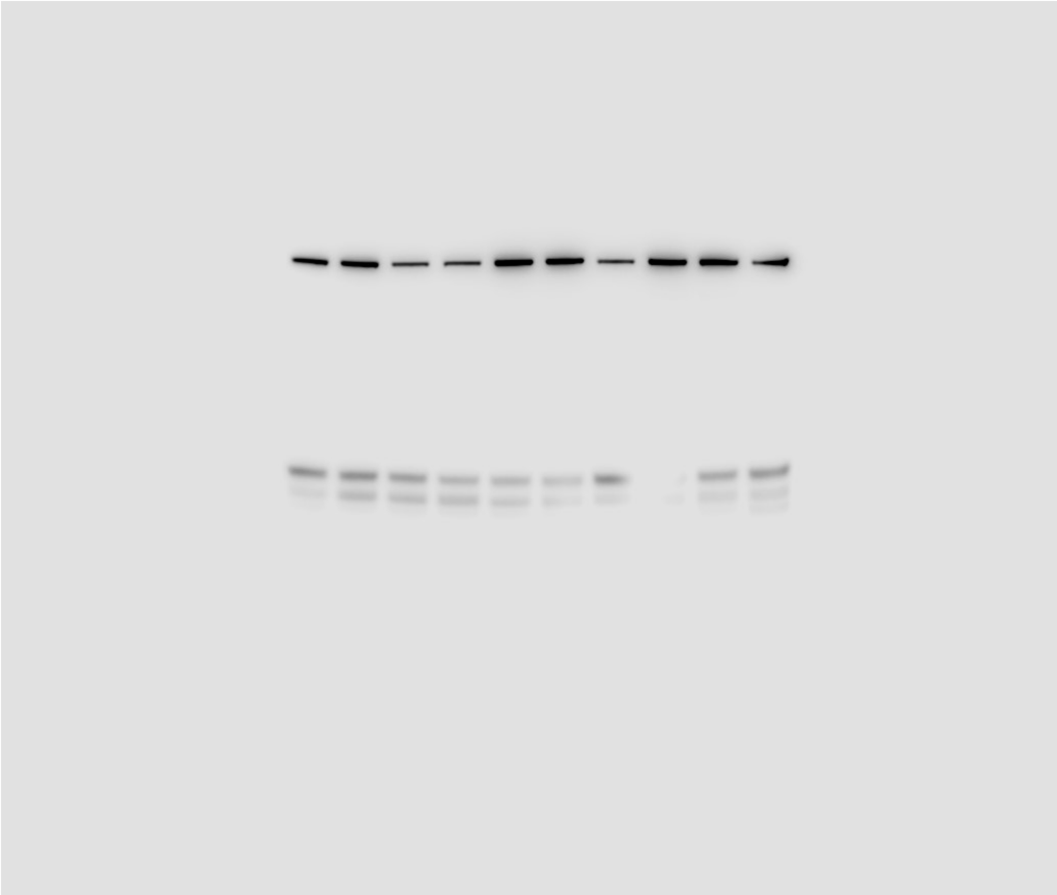

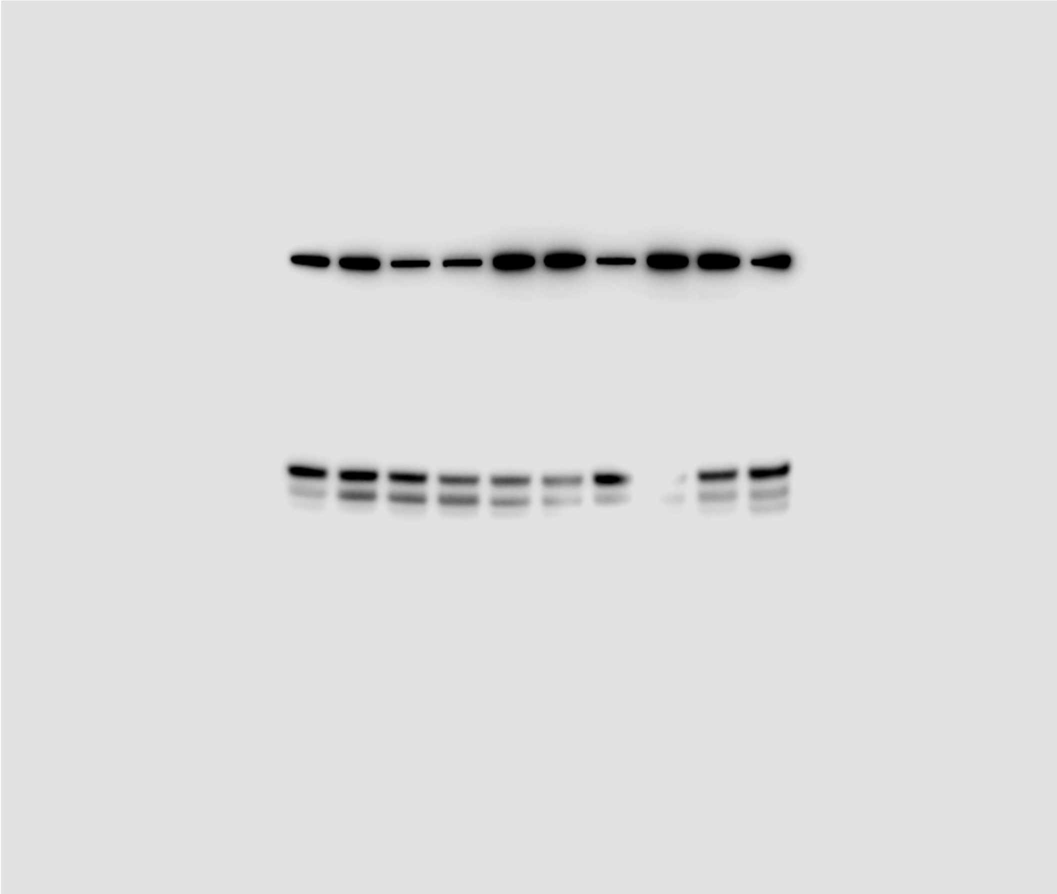

Figure 8B

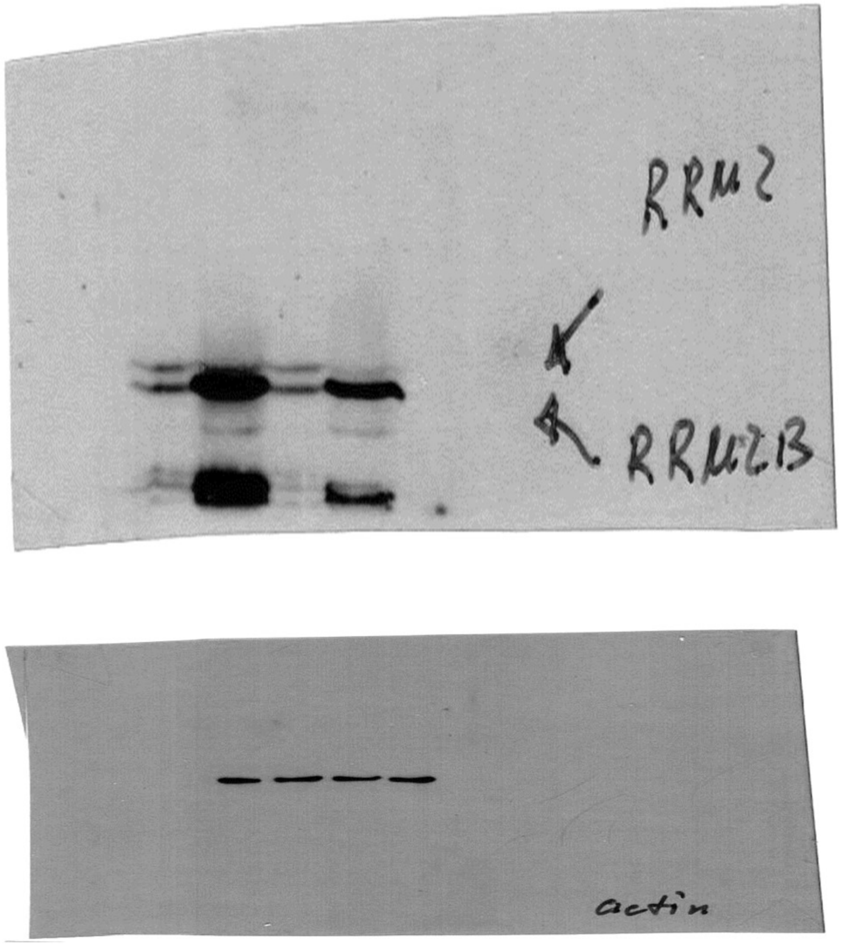

Suppl Figure 6B

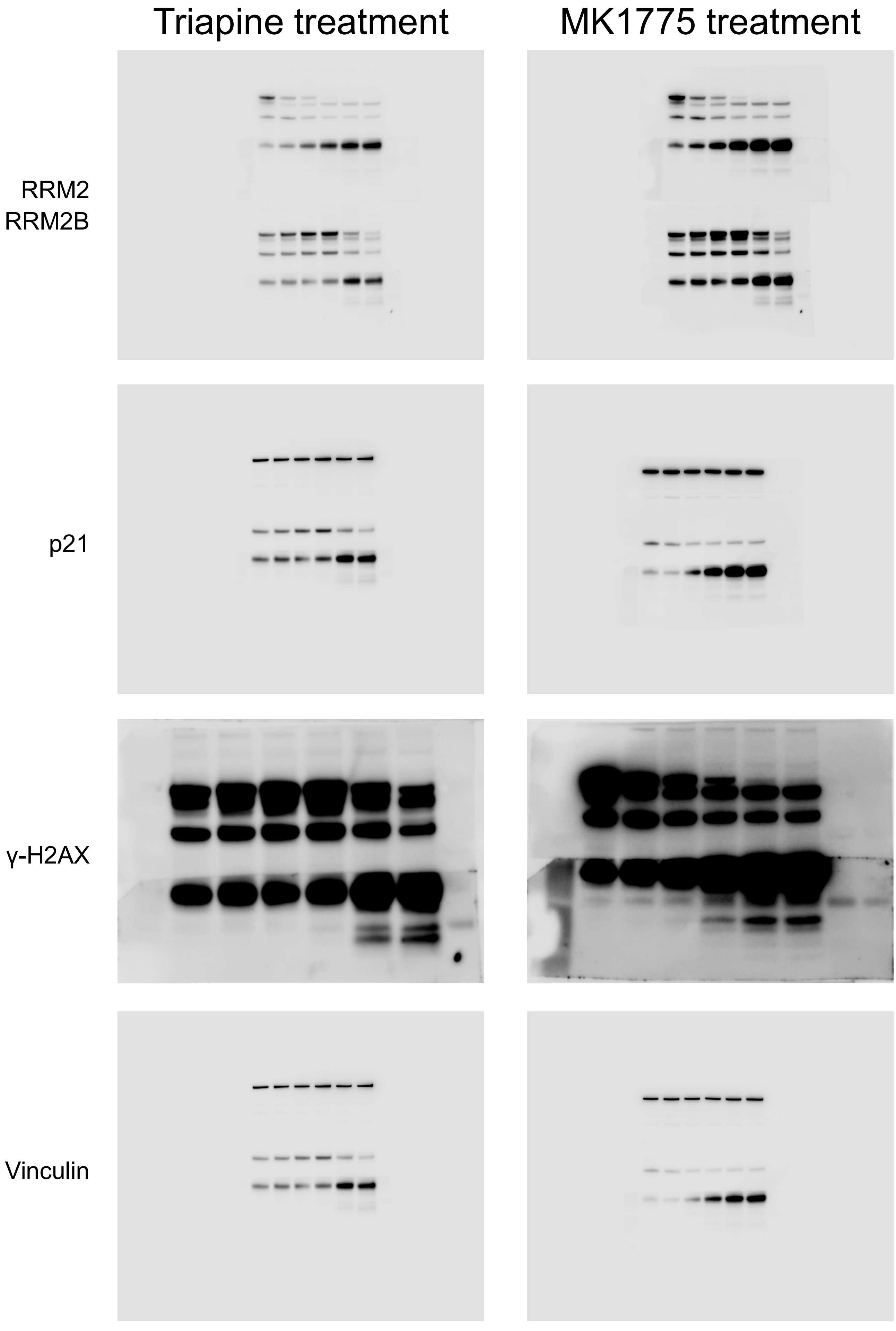

Suppl Figure S7B

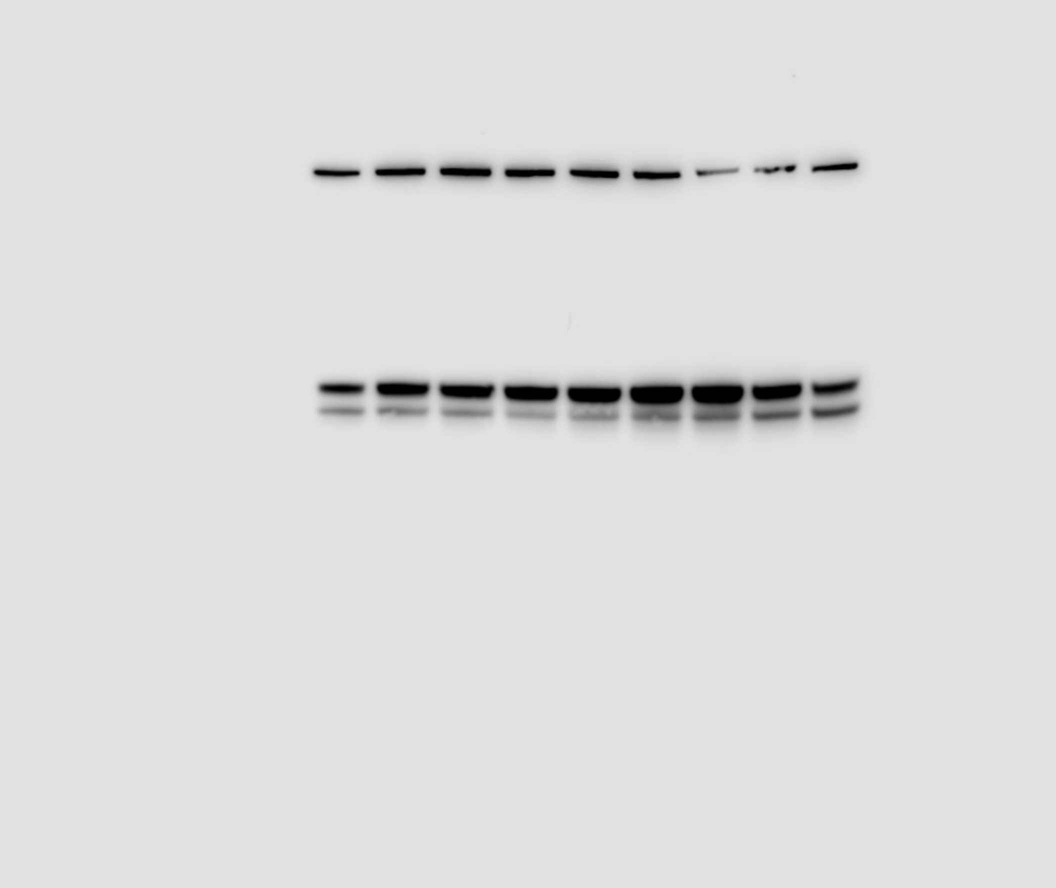

Suppl Figure S7C

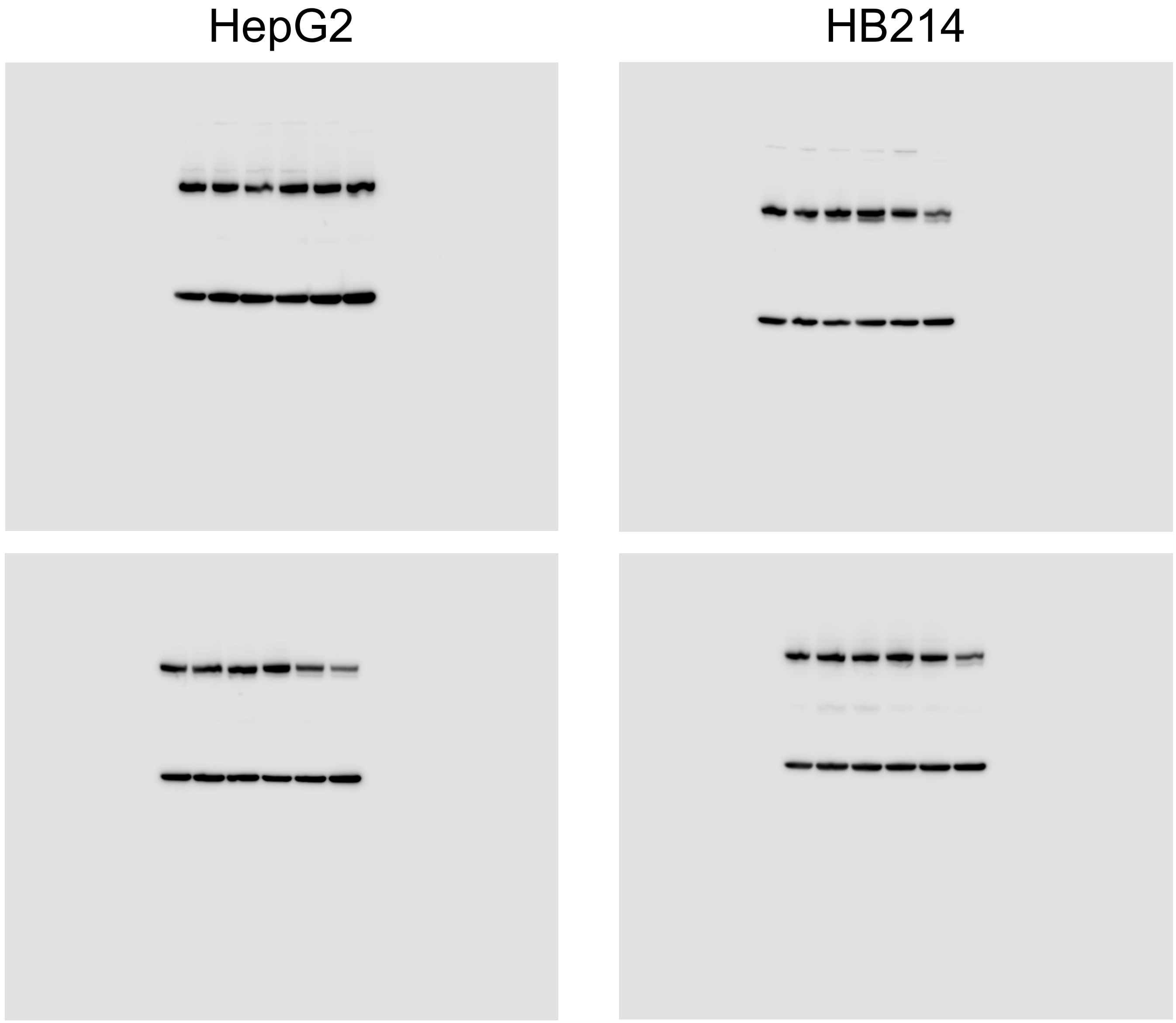

Suppl Figure S8

(Samples were run on the same gel in Figure 7A)

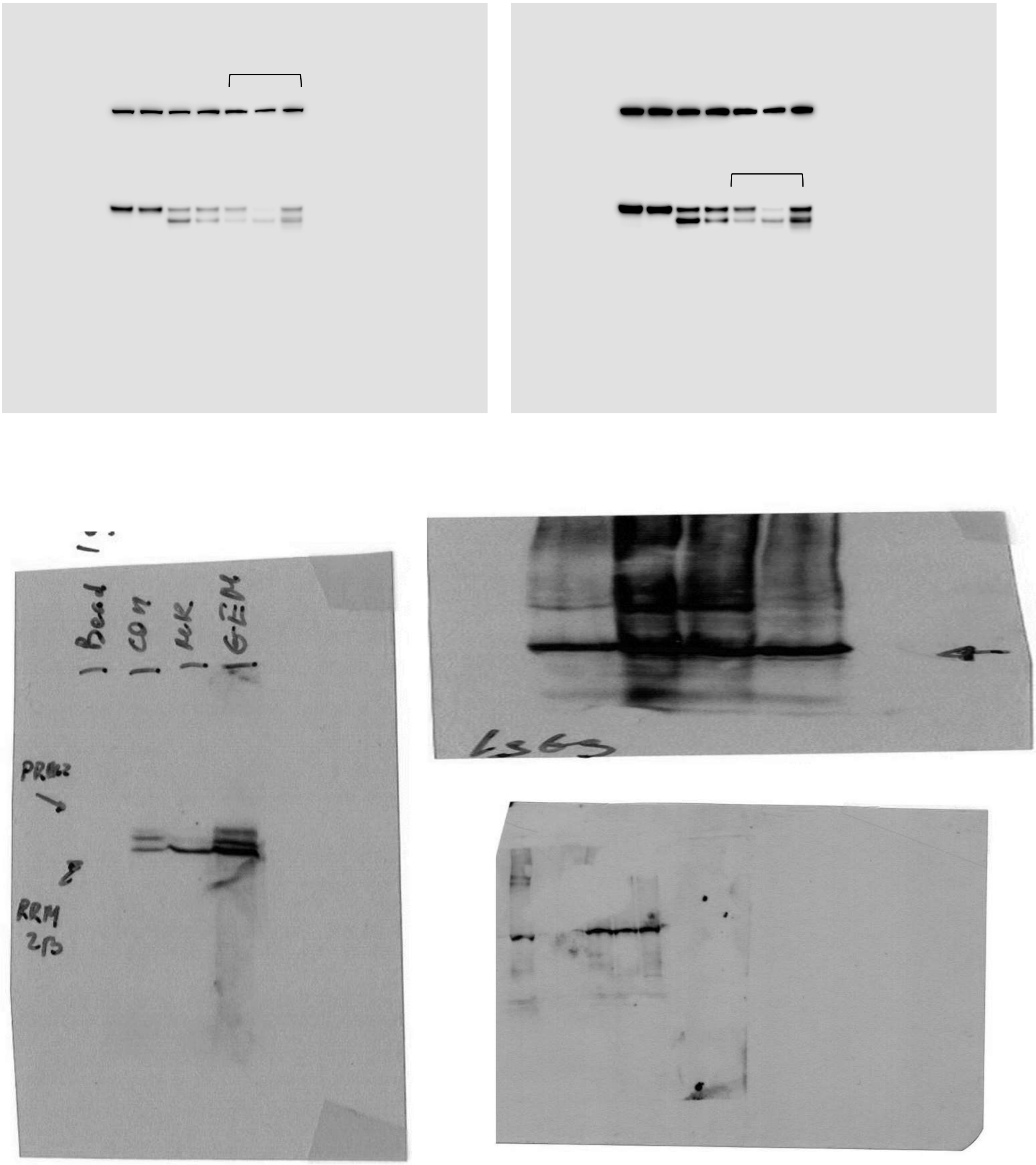

Suppl Figure S9A

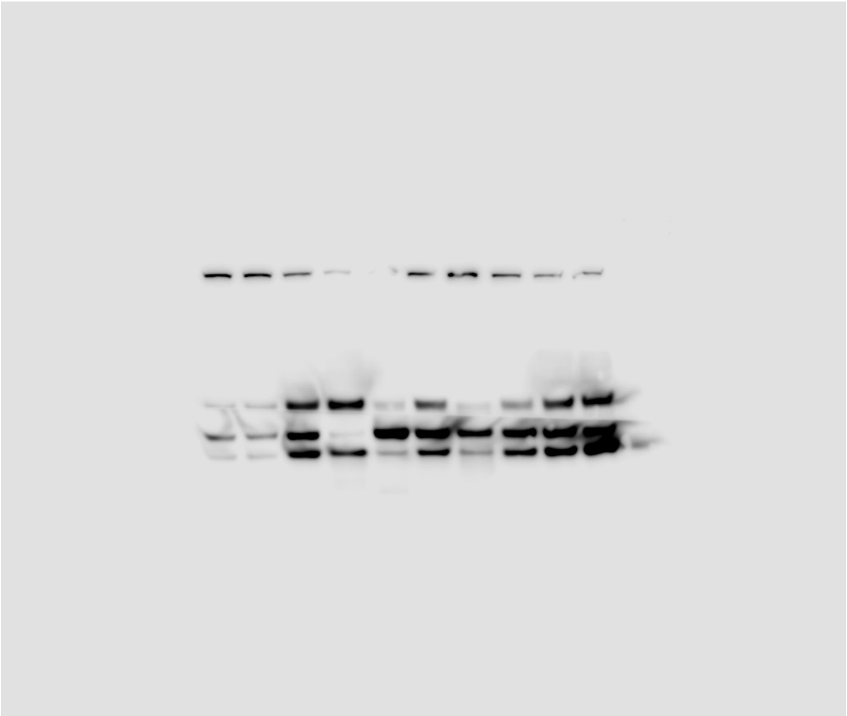

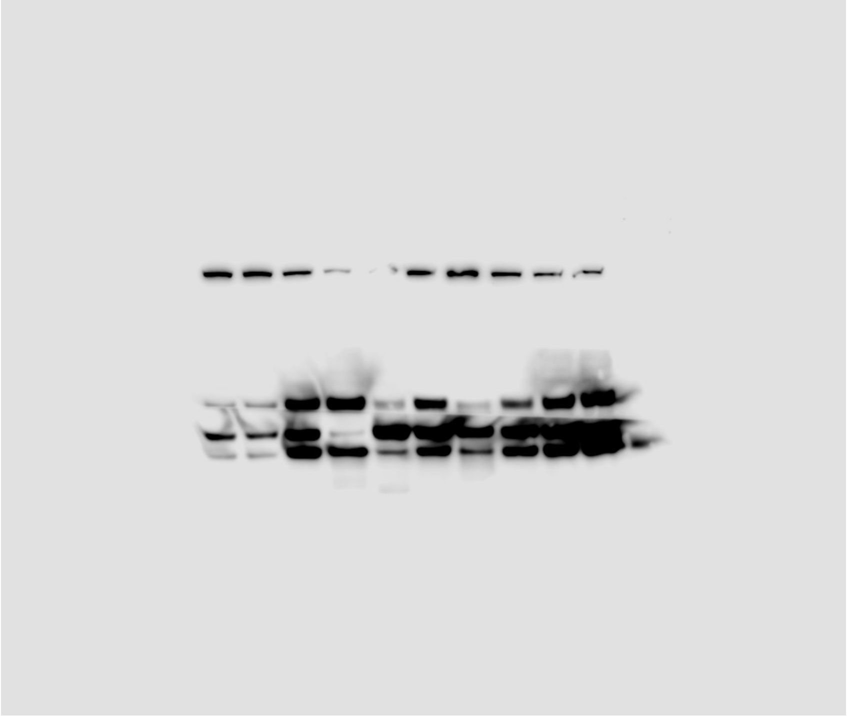

Suppl Figure S9B

Sorafenib Treatment

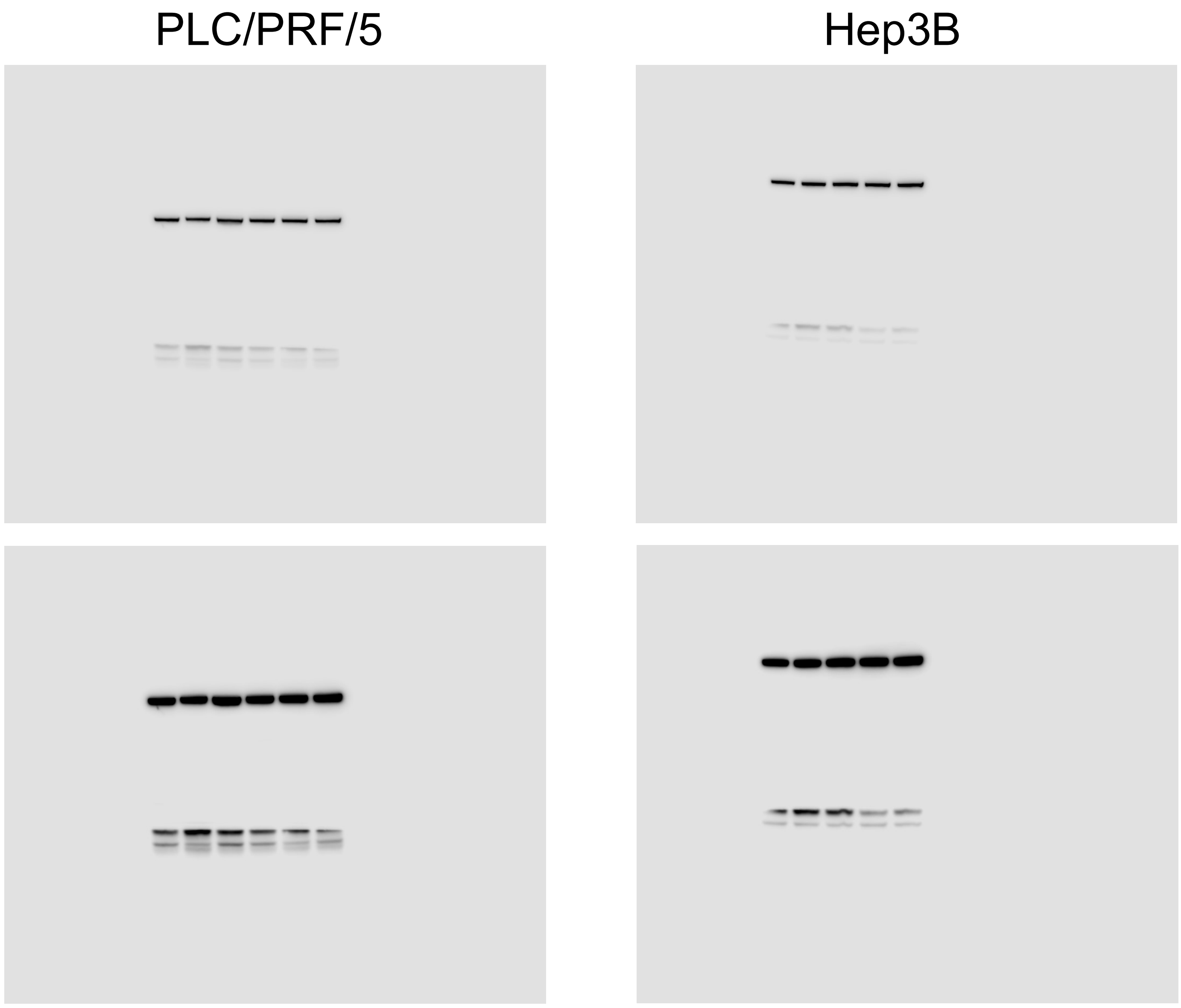

Suppl Figure S9B

Gemcitabine Treatment

PLC/PRF/5 Hep3B

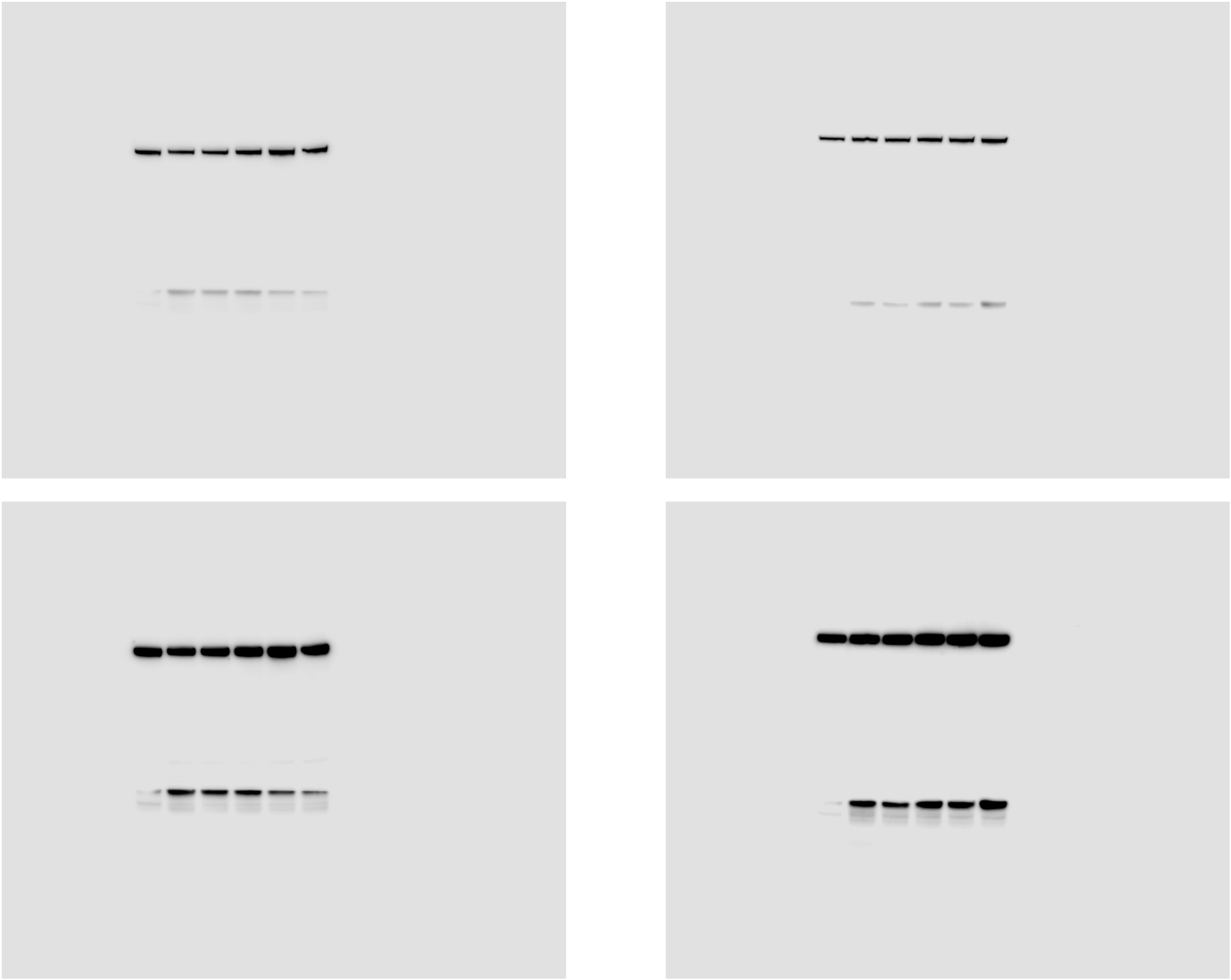

Suppl Figure S11

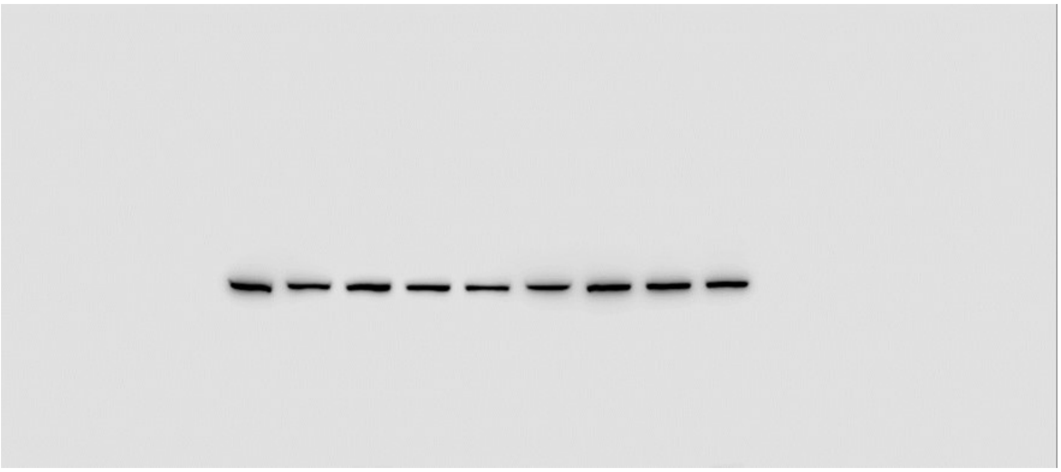

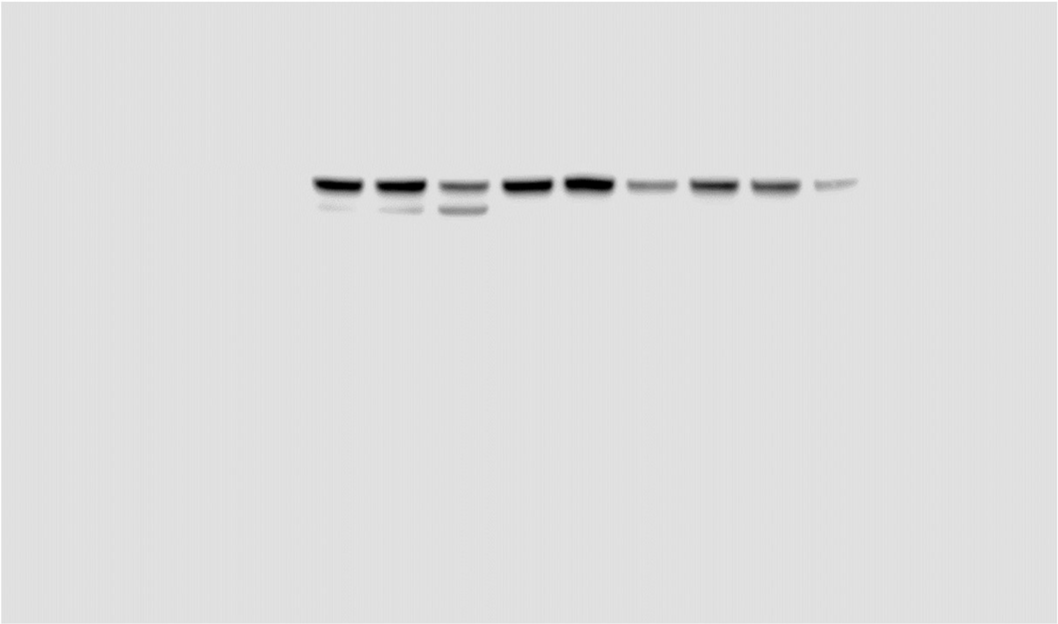

Suppl Figure S12

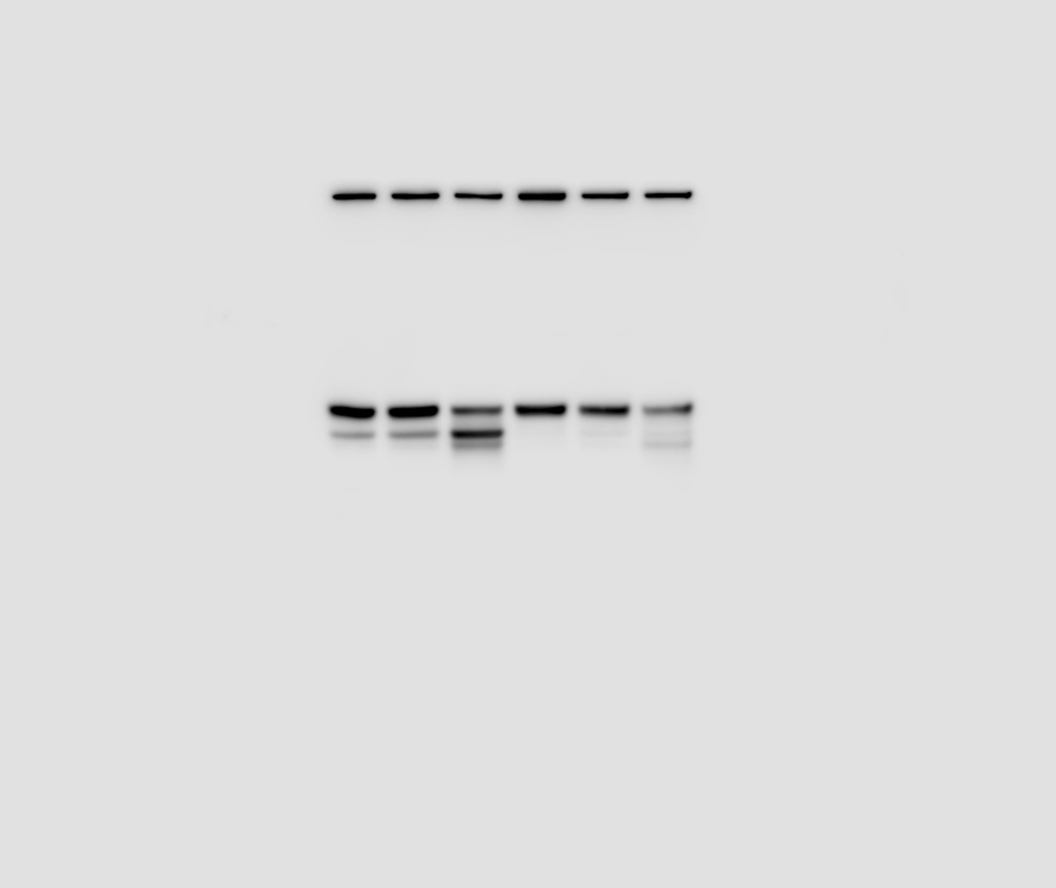

Suppl Figure S15

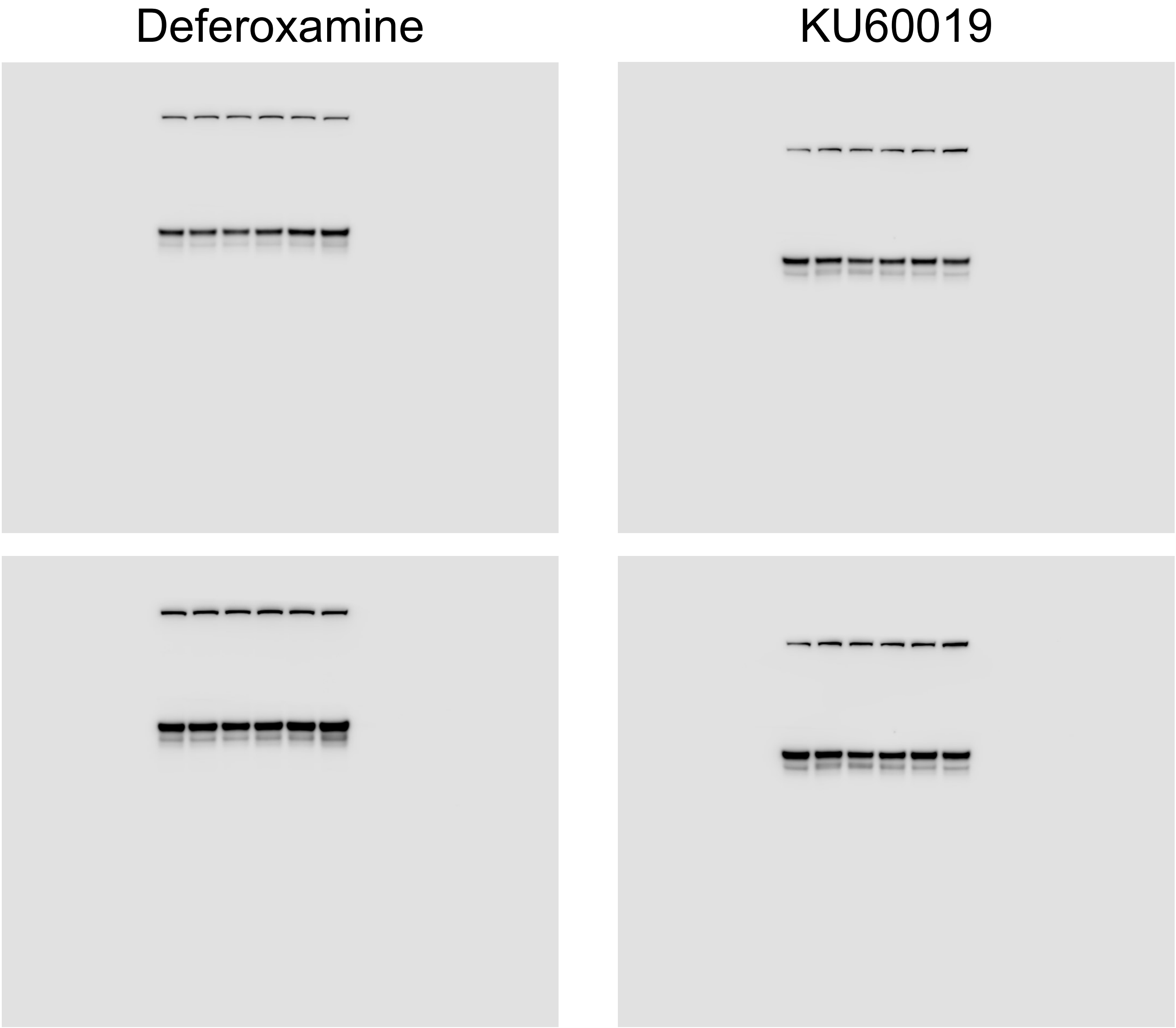

Supplement: Supplementary file 2 — Supplementary Information [file 42003_2023_4630_MOESM2_ESM.pdf]
