# Supplementary material for: Ribonucleotide reductase subunit switching in hepatoblastoma drug response and relapse
Source: Commun Biol. 2023 Mar 8;6:249. doi: 10.1038/s42003-023-04630-7 (PMC9992519; doi:10.1038/s42003-023-04630-7)
Supplement: Supplementary file 3 — Description of Additional Supplementary Files [file 42003_2023_4630_MOESM3_ESM.pdf]

Description of Additional Supplementary Files

File name: Supplementary Data 1

Description: The RRM2 and RRM2B hub gene lists

File name: Supplementary Data 2

Description: The numerical source data for graphs in Figure 3, 6 & 7

File name: Supplementary Data 3

Description: The quantification of all WB images (per the reviewer’s request)
