# Supplementary material for: Ribonucleotide reductase subunit switching in hepatoblastoma drug response and relapse
Source: Commun Biol. 2023 Mar 8;6:249. doi: 10.1038/s42003-023-04630-7 (PMC9992519; doi:10.1038/s42003-023-04630-7)
Supplement: Supplementary file 7 — Reporting Summary [file 42003_2023_4630_MOESM7_ESM.pdf]

nature portfolio

Corresponding author(s): Liqin Zhu

Last updated by author(s): Feb 13, 2023

Reporting Summary

Nature Portfolio wishes to improve the reproducibility of the work that we publish. This form provides structure for consistency and transparency in reporting. For further information on Nature Portfolio policies, see our Editorial Policies and the Editorial Policy Checklist.

Statistics

For all statistical analyses, confirm that the following items are present in the figure legend, table legend, main text, or Methods section.

n/a	Confirmed	
☐	☒	The exact sample size (n) for each experimental group/condition, given as a discrete number and unit of measurement
☐	☒	A statement on whether measurements were taken from distinct samples or whether the same sample was measured repeatedly
☐	☒	The statistical test(s) used AND whether they are one- or two-sided<br>*Only common tests should be described solely by name; describe more complex techniques in the Methods section.*
☒	☐	A description of all covariates tested
☒	☐	A description of any assumptions or corrections, such as tests of normality and adjustment for multiple comparisons
☒	☐	A full description of the statistical parameters including central tendency (e.g. means) or other basic estimates (e.g. regression coefficient) AND variation (e.g. standard deviation) or associated estimates of uncertainty (e.g. confidence intervals)
☒	☐	For null hypothesis testing, the test statistic (e.g. F, t, r) with confidence intervals, effect sizes, degrees of freedom and P value noted<br>*Give P values as exact values whenever suitable.*
☒	☐	For Bayesian analysis, information on the choice of priors and Markov chain Monte Carlo settings
☒	☐	For hierarchical and complex designs, identification of the appropriate level for tests and full reporting of outcomes
☒	☐	Estimates of effect sizes (e.g. Cohen's d, Pearson's r), indicating how they were calculated

Our web collection on statistics for biologists contains articles on many of the points above.

Software and code

Policy information about availability of computer code

Data collection	The details have been included in the manuscript Methods section for specific analyses.
Data analysis	The details have been included in the manuscript Methods section for specific analyses.

For manuscripts utilizing custom algorithms or software that are central to the research but not yet described in published literature, software must be made available to editors and reviewers. We strongly encourage code deposition in a community repository (e.g. GitHub). See the Nature Portfolio guidelines for submitting code & software for further information.

Data

Policy information about availability of data

All manuscripts must include a data availability statement. This statement should provide the following information, where applicable:

- Accession codes, unique identifiers, or web links for publicly available datasets
- A description of any restrictions on data availability
- For clinical datasets or third party data, please ensure that the statement adheres to our policy

The RNAseq datasets generated during this study are available in the NCBI GEO repository. The authors confirm that the data supporting the findings of this study are available within the article and its supplementary materials.

Human research participants

Policy information about studies involving human research participants and Sex and Gender in Research.

Reporting on sex and gender	No human research participants in this study.
Population characteristics	N/A
Recruitment	N/A
Ethics oversight	N/A

Note that full information on the approval of the study protocol must also be provided in the manuscript.

Field-specific reporting

Please select the one below that is the best fit for your research. If you are not sure, read the appropriate sections before making your selection.

☒ Life sciences ☐ Behavioural & social sciences ☐ Ecological, evolutionary & environmental sciences

For a reference copy of the document with all sections, see nature.com/documents/nr-reporting-summary-flat.pdf

Life sciences study design

All studies must disclose on these points even when the disclosure is negative.

Sample size	All experiments were repeated equal or more than three times independently and the results were compared to ensure statistically significance.
Data exclusions	No data were excluded in this study.
Replication	All experiments were repeated equal or more than three times independently.
Randomization	N/A
Blinding	No blinding was applied in this study as the phenotypes were not subjective.

Reporting for specific materials, systems and methods

We require information from authors about some types of materials, experimental systems and methods used in many studies. Here, indicate whether each material, system or method listed is relevant to your study. If you are not sure if a list item applies to your research, read the appropriate section before selecting a response.

Materials & experimental systems

n/a	Involved in the study	
☐	☒	Antibodies
☐	☒	Eukaryotic cell lines
☒	☐	Palaeontology and archaeology
☐	☒	Animals and other organisms
☒	☐	Clinical data
☒	☐	Dual use research of concern

Methods

n/a	Involved in the study	
☒	☐	ChIP-seq
☒	☐	Flow cytometry
☒	☐	MRI-based neuroimaging

Antibodies

Antibodies used	anti-p53R2 + RRM2 antibody (abcam, Cambridge, United Kingdom, ab209995, 1:10000), anti-RRM2 antibody (abcam, ab172476, 1:5000), phospho-NF-κB p65 (Ser536)(93H1) Rabbit mAb (CST #3033, 1:1000), NF-κB p65(D14E12)XP Rabbit antibody (CST #8242, 1:1000), Phospho-AMPKα (Thr172) antibody (CST #2531, 1:1000), p53 (7F5) Rabbit mAb(CST #2527, 1:1000). anti-Ki67 (Abcam, Cambridge, MA, USA, ab16667, 1:200)
Validation	All antibodies used are commercial antibodies that have been widely used and reported in the field of cancer research.

Eukaryotic cell lines

Policy information about cell lines and Sex and Gender in Research

Cell line source(s)	HepG2: commercial human cell line purchased from ATCC<br>HB214: primary cell line derived from a hepatoblastoma PDX model
Authentication	HepG2 was authenticated by short tandem repeat (STR) profiling; HB214 has no public STR profile. This cell line was authenticated via the hepatoblastoma tumors it generates in NSG mouse liver.
Mycoplasma contamination	Our lab performed mycoplasma test regularly and both cell lines are negative for mycoplasma.
Commonly misidentified lines<br>(See ICLAC register)	Neither cell line is a commonly misidentified line.

Animals and other research organisms

Policy information about studies involving animals; ARRIVE guidelines recommended for reporting animal research, and Sex and Gender in Research

Laboratory animals	two-month-old NSG (NOD scid gamma) mice
Wild animals	No wild animals used in the study.
Reporting on sex	Both male and female mice are used in the study.
Field-collected samples	No field-collected samples are used in the study.
Ethics oversight	Animal protocols were approved by the St. Jude Children's Research Hospital Animal Care and Use Committee.

Note that full information on the approval of the study protocol must also be provided in the manuscript.
